# A macrocyclic kinase inhibitor overcomes triple resistant mutations in EGFR-positive lung cancer

**DOI:** 10.1038/s41698-024-00542-9

**Published:** 2024-02-23

**Authors:** Mai Suzuki, Ken Uchibori, Tomoko Oh-hara, Yumi Nomura, Ryusei Suzuki, Ai Takemoto, Mitsugu Araki, Shigeyuki Matsumoto, Yukari Sagae, Mutsuko Kukimoto-Niino, Yusuke Kawase, Mikako Shirouzu, Yasushi Okuno, Makoto Nishio, Naoya Fujita, Ryohei Katayama

**Affiliations:** 1 Division of Experimental Chemotherapy, Cancer Chemotherapy Center, Japanese Foundation for Cancer Research, Tokyo, 135-8550 Japan; 2 Department of Medical Genome Science, Graduate School of Frontier Science, The University of Tokyo, Tokyo, 108-8639 Japan; 3 Department of Thoracic Medical Oncology, The Cancer Institute Hospital, Japanese Foundation for Cancer Research, Tokyo, Japan; 4 Graduate School of Medicine, Kyoto University, 53 Shogoin-Kawaharacho, Sakyo-Ku, Kyoto, Japan; 5 Laboratory for Protein Functional and Structural Biology, RIKEN Center for Biosystems Dynamics Research, Yokohama, Kanagawa 230-0045 Japan; 6 Carna Biosciences, Inc, Kobe, 650-0047 Japan; 7 Cancer Chemotherapy Center, Japanese Foundation for Cancer Research, Tokyo, Japan

**Keywords:** Non-small-cell lung cancer, Non-small-cell lung cancer, Cancer therapeutic resistance

## Abstract

Brigatinib-based therapy was effective against osimertinib-resistant EGFR C797S mutants and is undergoing clinical studies. However, tumor relapse suggests additional resistance mutations might emerge. Here, we first demonstrated the binding mode of brigatinib to the EGFR-T790M/C797S mutant by crystal structure analysis and predicted brigatinib-resistant mutations through a cell-based assay including N-ethyl-N-nitrosourea (ENU) mutagenesis. We found that clinically reported L718 and G796 compound mutations appeared, consistent with their proximity to the binding site of brigatinib, and brigatinib-resistant quadruple mutants such as EGFR-activating mutation/T790M/C797S/L718M were resistant to all the clinically available EGFR-TKIs. BI-4020, a fourth-generation EGFR inhibitor with a macrocyclic structure, overcomes the quadruple and major EGFR-activating mutants but not the minor mutants, such as L747P or S768I. Molecular dynamics simulation revealed the binding mode and affinity between BI-4020 and EGFR mutants. This study identified potential therapeutic strategies using the new-generation macrocyclic EGFR inhibitor to overcome the emerging ultimate resistance mutants.

## Introduction

Approximately 30–40% of Japanese non-small cell lung cancer patients have mutations in epidermal growth factor receptor (EGFR)^1^. It is known that approximately 90% of these patients have del19 mutations, in which 3–7 amino acids in the exon19 encoding N-terminal lobe of the tyrosine kinase domain of EGFR are deleted, or L858R mutations, which are point mutations in exon21 (major mutations)^2^. The remaining 10% of EGFR mutants consist of minor mutations, such as G719X in exon18, L747P in exon19, 1–4 amino acid insertion mutation or S768I of exon20, and L861Q of exon21^2,3^. All of these mutations are known as EGFR-activating mutations that induce ligand-independent EGFR activation, and recent studies have revealed in detail their structures and sensitivity to various EGFR inhibitors^4^.

To date, several EGFR-tyrosine kinase inhibitors (EGFR-TKIs) have been developed worldwide for the treatment of EGFR-activating mutations, and at least five drugs are widely used in clinical settings^5–7^. In the past, first-generation EGFR inhibitors, gefitinib and erlotinib, and second-generation EGFR inhibitors, afatinib, and dacomitinib, have been used as first-line therapy and have shown high efficacy, especially against del19 and L858R mutants^8,9^. However, it has been reported that within a few years, most patients acquire resistance to these drugs, especially 50–70% of the patients acquired T790M resistance mutation in the ATP-binding pocket, known as a gatekeeper mutation^10^. The third-generation EGFR inhibitor osimertinib has been developed as a drug that can overcome the T790M mutant and has demonstrated a high response rate^11,12^. Despite this, ~10–20% of patients develop resistance to osimertinib because of the additional C797S mutation, which occurs at a cysteine residue required for covalent binding of osimertinib, and the resultant EGFR compound mutant (activating mutation/T790M/C797S) showed marked resistance to all EGFR inhibitors^13–15^. In 2018, the efficacy of osimertinib in first-line therapy was also reported; hence, it was approved for use as first-line therapy^16^. Nevertheless, ~10% of patients still develop resistance to osimertinib due to the emergence of the C797S mutation^17^. For EGFR-activating mutation/C797S compound mutants, gefitinib and afatinib were experimentally shown to be effective, but eventually, the T790M mutation might be acquired. Thus, the development of drugs to overcome the EGFR-activating mutation/T790M/C797S mutant is needed^18^.

In 2017, we reported that combination therapy with the ALK inhibitor brigatinib and anti-EGFR antibody was effective against the EGFR-activating mutation/T790M/C797S and EGFR-activating mutation/C797S mutants^19^. Based on these results, a phase I/II clinical trial of brigatinib + panitumumab in C797S mutation-positive non-small cell lung cancer after osimertinib treatment has been ongoing since 2020. Since 2019, several clinical studies on the combination of brigatinib and an anti-EGFR antibody have been reported by Chinese researchers^20–22^. In 2020, Wang et al. reported that the combination of brigatinib and anti-EGFR antibody cetuximab showed favorable results, with PFS reaching 14 months and an overall response rate of 60%^21^. However, the efficacy of brigatinib in comparison with conventional chemotherapy has not yet been evaluated in a sufficient number of patients, and further investigation of the efficacy of brigatinib therapy is needed. Recently, fourth-generation EGFR inhibitors, such as EAI-045, BLU-945, JBJ-04-125-02, and BI-4020, have been developed to overcome the T790M/C797S compound mutant^23–27^. One of these, BLU-945, is currently being used in clinical trials. The efficacy of these novel therapeutic candidates in the treatment of the T790M/C797S compound mutant should be further investigated in the future.

While the efficacy of brigatinib with anti-EGFR antibody therapy is currently under investigation, new mutations resistant to brigatinib therapy are also expected to emerge. In this study, we first determined the binding mode of brigatinib with mutant EGFR using crystal structure analysis. Moreover, we predicted resistance mutations to brigatinib therapy through saturation mutagenesis screening and explored drugs to overcome such resistance mutants.

## Results

### Brigatinib with EGFR antibody effectively inhibits the tumor growth of EGFR-activating mutation/C797S or EGFR-activating mutation/T790M/C797S mutants in vitro and in vivo

**Fig. 1:**
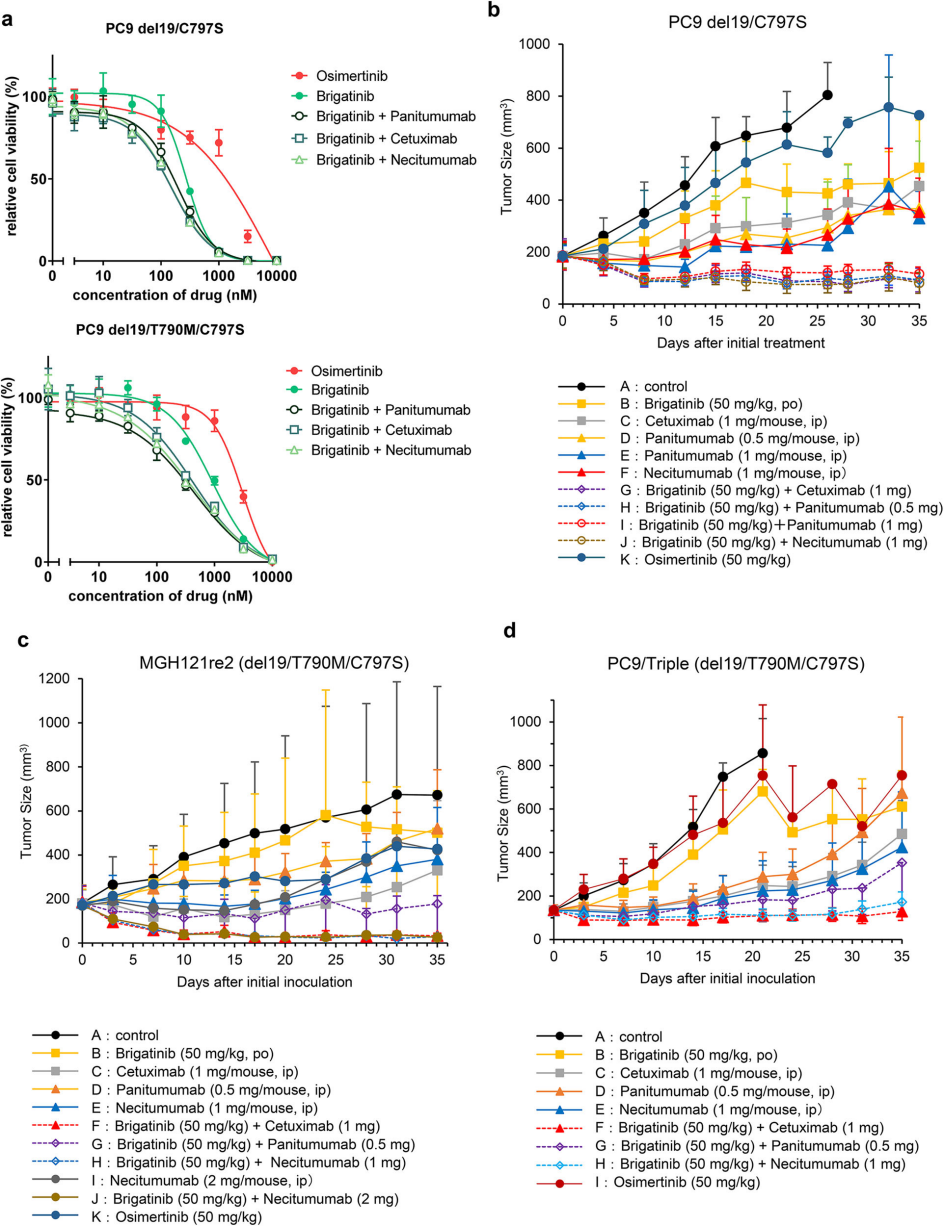
Brigatinib with anti-EGFR antibody therapy effectively inhibits osimertinib-resistant tumor growth in vitro and in vivo. **a** PC9 cells expressing EGFR-del19/T790M/C797S or EGFR-del19/C797S were treated with serially diluted brigatinib with cetuximab, panitumumab, or necitumumab for 72 h. After drug treatment, the cell viability was evaluated by the CellTiter-Glo assay. Relative cell viability was presented as mean ± SD. **b**–**d** PC9-del19/C797S cells (**b**), MGH121re2 cells (**c**), or PC9-del19/T790M/C797S mutant cells (d) were subcutaneously injected into nude mice, and treated with the indicated doses of brigatinib with or without various anti-EGFR antibodies.

To examine the efficacy of brigatinib with EGFR antibodies in vitro and in vivo, PC9 cells expressing EGFR-del19/C797S or EGFR-del19/T790M/C797S were treated with brigatinib and various EGFR antibodies, cetuximab, panitumumab, or necitumumab. As previously reported^19^, any of the EGFR antibodies with brigatinib effectively inhibited the growth of osimertinib-resistant EGFR mutants (Fig. 1a). In contrast, osimertinib with EGFR antibody could not inhibit the growth of C797S harboring EGFR mutant in Ba/F3 model (Supplementary Fig. 1). Next, to evaluate the efficacy of brigatinib with EGFR antibody therapy in vivo, PC9 expressing EGFR-del19/C797S, MGH121re2, which harbors endogenous EGFR-del19/T790M/C797S, or PC9 expressing EGFR-del19/T790M/C797S mutants were subcutaneously inoculated into immunodeficient mice and treated with 50 mg/kg of brigatinib with 0.5 or 1 mg cetuximab, panitumumab, or necitumumab (Fig. 1b–d). The results demonstrated that the combination therapies induced marked tumor growth suppression for more than 1 month, suggesting that brigatinib with any of the anti-EGFR antibodies is effective against osimertinib-resistant EGFR-del19/C797S or -del19/T790M/C797S tumors.

### Crystal structure analysis of brigatinib complexed with EGFR-T790M/C797S

**Fig. 2:**
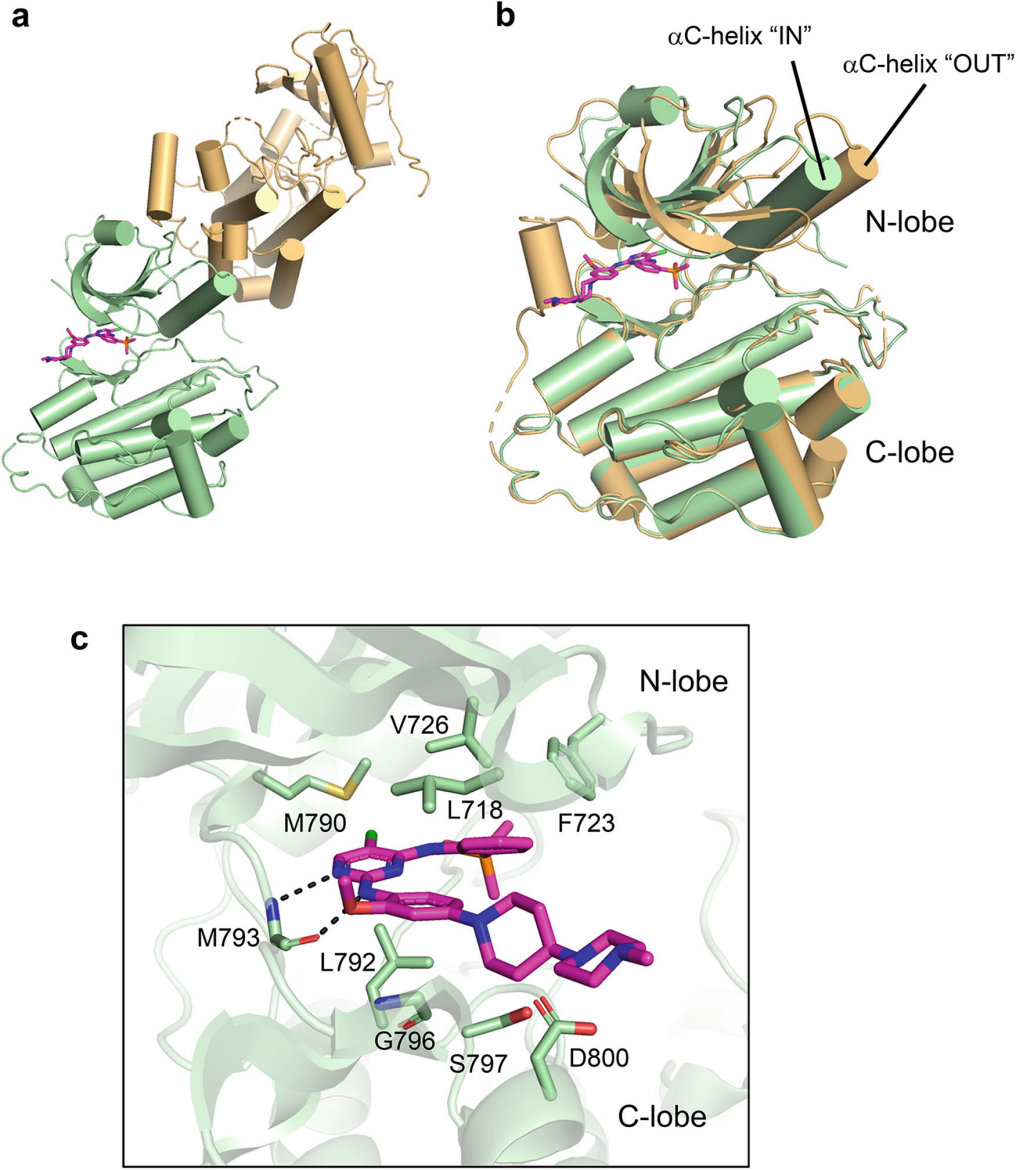
Crystal structure analysis of brigatinib complexed with EGFR kinase domain with T790M and C797S mutations. **a** Overall structure of the asymmetric dimer of the EGFR-T790M/C797S kinase domain. The receiver (brigatinib-bound form) and activator (apo-form) subunits of the dimer are colored green and orange, respectively. Brigatinib is shown as a magenta stick model. The structure data was deposited at PDB with ID: 8H7X. **b** Comparison of the structures of brigatinib-bound EGFR-T790M/C797S (green) and apo-form EGFR-T790M/C797S (orange). The “in” and “out” conformations of the brigatinib-bound and apo-form αC-helices correspond to the active and inactive conformations of the EGFR kinase domain, respectively. **c** Binding mode of brigatinib to EGFR-T790M/C797S. Brigatinib (magenta) and selected residues of EGFR-T790M/C797S (green) are shown as stick models.

Since brigatinib directly inhibits EGFR mutant kinases^19^, to understand how brigatinib binds to EGFR-T790M/C797S mutant, we determined the crystal structure of the T790M/C797S mutant EGFR tyrosine kinase domain in complex with brigatinib (Supplementary Table 1). EGFR-T790M/C797S formed an asymmetric dimer composed of the donor and receiver subunits, exhibiting inactive and active conformations, respectively (Fig. 2a and b). Brigatinib bound only to the receiver subunit of the EGFR kinase domain, and the brigatinib-bound form of EGFR-T790M/C797S was quite similar to the structural model we previously predicted by molecular dynamics (MD) simulations^19^. Brigatinib formed two hydrogen bonds with the main chain of M793 and was in contact with many hydrophobic residues such as L718, L792, and F723. In contrast, the M790 and S797 residues, which are the main causes of resistance to the existing EGFR-TKIs, were slightly distant from brigatinib (Fig. 2c). These results indicated that brigatinib preferentially binds to the active conformation of the receiver subunit of the asymmetric EGFR kinase dimer.

### Identification of brigatinib-resistant EGFR mutations by ENU mutagenesis screening

**Fig. 3:**
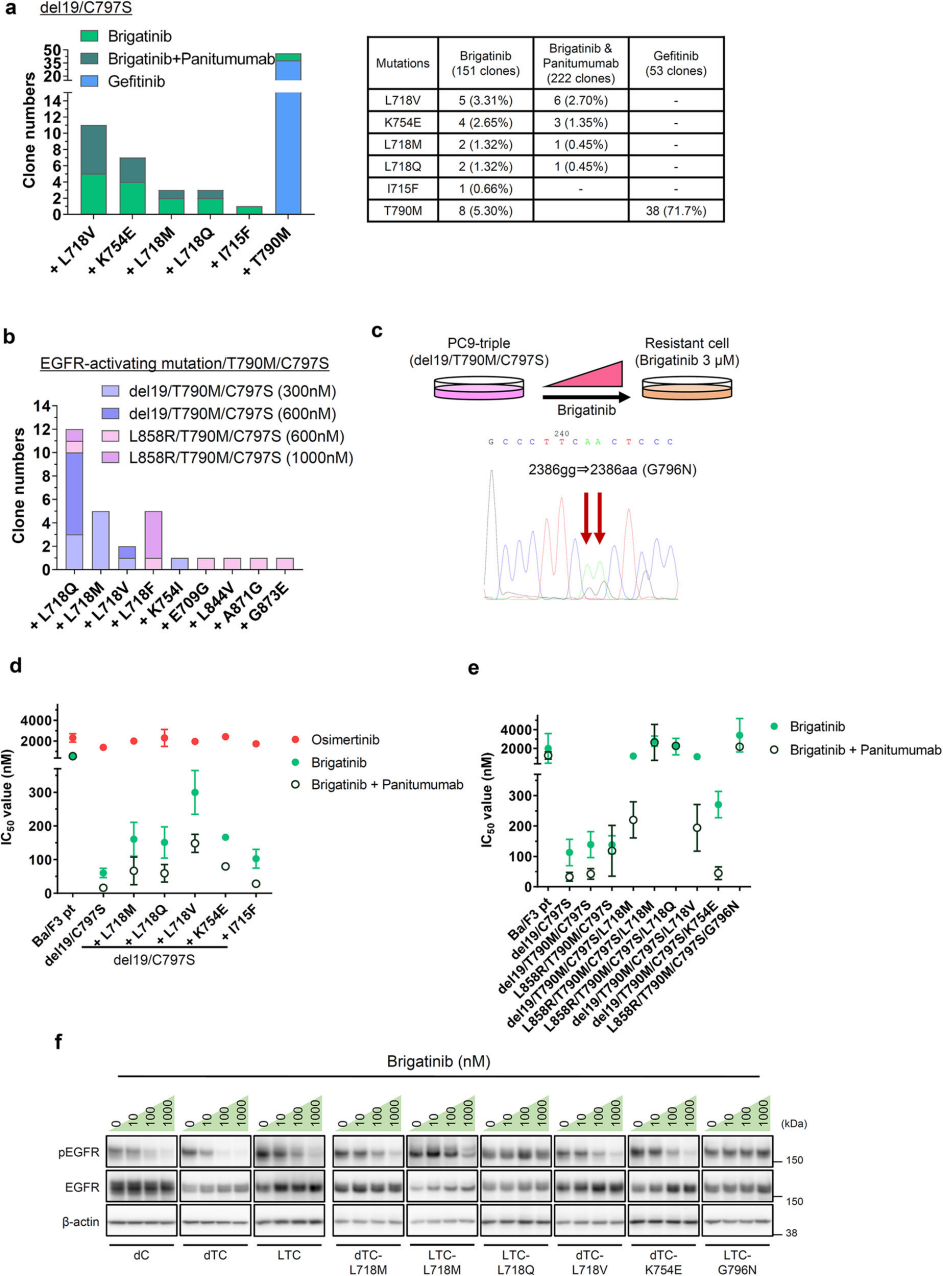
ENU mutagenesis screening identified brigatinib-resistant mutations using Ba/F3-EGFR-C797S or Ba/F3-EGFR-T790M/C797S positive cells. **a**, **b** The number of clones obtained from ENU mutagenesis screening. **a** L718, K754, and I715 mutations were obtained from Ba/F3-del19/C797S treated with brigatinib or brigatinib + panitumumab. **b** L718, K754, E709, L844, A871, and G873 mutations were obtained from Ba/F3-del19 or L858R (activating mutation)/T790M/C797S cells treated with brigatinib (300, 600, or 1000 nM). **c** G796N mutation was obtained from PC9-del19/T790M/C797S cells continuously treated with brigatinib. **d**, **e** IC_50_ values of brigatinib, brigatinib + panitumumab, and osimertinib in mutants obtained from ENU mutagenesis. Ba/F3-del19/C797S/+ each mutation expressing cells (**d**) and Ba/F3-del19/T790M/C797S/+ each mutation expressing cells (**e**). Cells were treated with osimertinib, brigatinib, and brigatinib + panitumumab for 72 h.; *N* = 3 or 6. Results are presented as mean ± SD. **f** Western blotting analysis of Ba/F3 cells expressing each mutation. Cells were treated with the indicated concentration of brigatinib for 3 h. d del19; L L858R; C C797S; T T790M. Uncropped immunoblot images are presented in Supplementary Fig. 10.

To predict the resistance mutations that might emerge after brigatinib with anti-EGFR antibody therapy, we performed ENU saturation mutagenesis screening using Ba/F3-del19/C797S, -del19 (or L858R)/T790M/C797S cells. After introducing mutations by ENU treatment, the cells were cultured in the presence of gefitinib (positive control), brigatinib, or brigatinib with an anti-EGFR antibody (Fig. 3a). As a result, The T790M mutation, a well-known gefitinib or erlotinib-resistant mutation, was observed in 38 of the 53 clones (71.7%) from the gefitinib-treated group, confirming that the mutagenesis screening was adequate, and Ba/F3 cells harboring brigatinib-resistant mutations were generated as expected (Fig. 3a). In Ba/F3-del19/C797S, the addition of each L718V, K754E, L718M, L718Q, and I715F mutation was identified in the brigatinib single treatment group. L718V, K754E, L718M, and L718Q were also detected in the brigatinib and panitumumab combination treatment groups (Fig. 3a). Mutagenesis screening using Ba/F3-del19/T790M/C797S cells revealed L718Q/M/V and K754I mutations in brigatinib single treatment (Fig. 3b). In the Ba/F3-L858R/T790M/C797S cells, the addition of L718Q/F, E709G, L844V, A871G, and G873E was identified in the brigatinib-treated group (Fig. 3b).

Furthermore, the G796N mutation was obtained by the continuous treatment with brigatinib to PC9-del19/T790M/C797S cells (Fig. 3c).

To confirm whether these compound mutants induced brigatinib resistance, Ba/F3 clones expanded directly from surviving cells in ENU mutagenesis screening or reconstructed Ba/F3-EGFR-compound mutant cells were examined for drug sensitivity using brigatinib or EGFR inhibitors. We confirmed that Ba/F3-del19/C797S and all Ba/F3-del19/C797S/+ each L718M/Q/V, I715F, or K754E mutation-expressing cells were resistant to osimertinib (Fig. 3d). Compared with Ba/F3-del19/C797S mutant cells, the addition of each L718M/Q/V, I715F, and K754E mutation resulted in a 1.7- to 4.9-fold increase in the IC50 values of the single treatment with brigatinib. In the combination treatment of brigatinib and panitumumab, the I715F compound mutant showed an IC_50_ value similar to that of the del19/C797S mutant, but the addition of each L718M/Q/V and K754E mutation to del19/C797S increased the IC_50_ values more than twice (Fig. 3d).

Similarly, compared with the IC_50_ value of the EGFR-activating mutation/T790M/C797S in brigatinib single treatment, the addition of each L718M/Q/V, K754E, and G796N mutation resulted in a 1.7- to 21-fold increase in IC_50_ values (Fig. 3e). Additionally, in combination with panitumumab, the K754E mutant did not significantly increase the IC50 value compared with that of the EGFR-activating mutation/T790M/C797S, but the addition of each L718M/Q/V, K754E, and G796N mutation significantly increased the IC50 value from 1.7-to 18-fold (Fig. 3e). Western blotting analysis showed that the phosphorylation level of EGFR in del19/C797S and del19/T790M/C797S mutants was markedly reduced at 100 nM brigatinib treatment. No significant reduction in phosphorylation level was observed with the addition of each L718M/Q/V, K754E, and G796N mutation, indicating that these compound mutants are resistant to brigatinib (Fig. 3f).

### BI-4020 overcomes brigatinib-resistant compound mutants

**Fig. 4:**
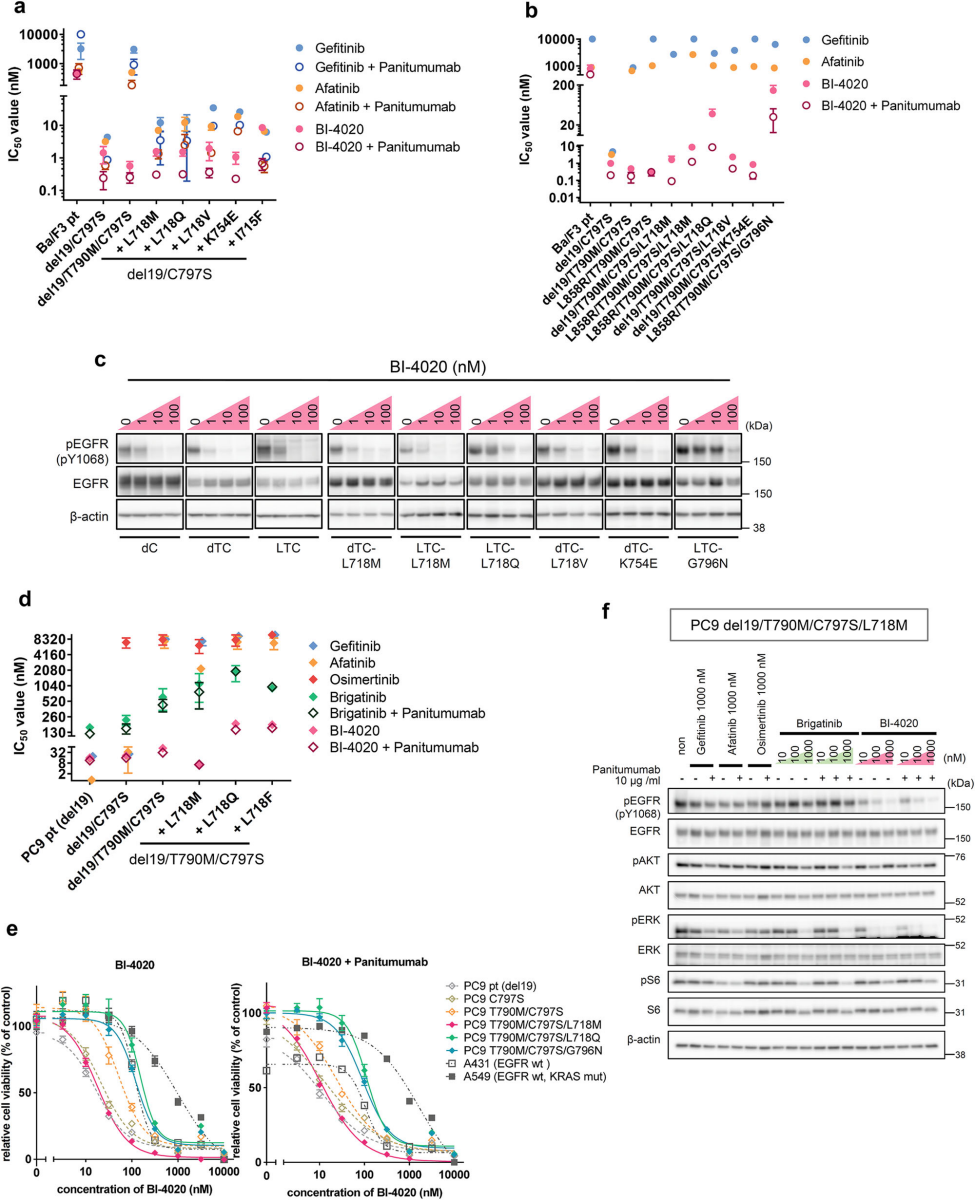
BI-4020 overcomes brigatinib-resistant compound mutants. **a**, **b** IC_50_ values were calculated from the cell viability assay data using Ba/F3 cells expressing indicated EGFR mutants. Brigatinib-resistant triple (**a**) and brigatinib-resistant quadruple (**b**) mutant cells were incubated in the medium containing serially diluted gefitinib, afatinib, or BI-4020 with or without 10 µg/ml of panitumumab for 72 h. Calculated IC_50_s are presented. For BI-4020 ± Panitumumab treatment, mean IC_50_s ± SD are presented. **c** Western blotting of Ba/F3 cells expressing each mutation. Cells were treated with the indicated concentration of BI-4020 for 3 h. d del19; L L858R; C C797S; T T790M. Uncropped immunoblot images are presented in Supplementary Fig. 10. **d** IC_50_ values calculated from the cell viability assay data using PC9 lung cancer cell line models. Brigatinib-resistant quadruple mutant cells were treated with each drug for 72 h. Results are presented as mean ± SD. **e** The selectivity of BI-4020 and combination treatment with panitumumab using A431 and A549 cell lines. Cells were treated with each drug for 72 h. **f** Western blotting of PC9-del19/T790M/C797S/L718M. Cells were treated with the indicated concentration of each drug for 6 h, and the cell lysates were analyzed by immunoblotting with the indicated antibodies. Uncropped immunoblot images are presented in Supplementary Fig. 10.

To overcome the brigatinib-resistant triple or quadruple compound mutants described above, we searched for effective therapeutic candidates, mainly EGFR-TKIs and ERBB family inhibitors. As previously reported^18^, the first- and second-generation EGFR inhibitors, gefitinib and afatinib, were ineffective in Ba/F3 cells expressing EGFR-del19/T790M/C797S, whereas Ba/F3-del19/C797S and brigatinib-resistant triple mutants showed high sensitivity to gefitinib and afatinib (Fig. 4a). Panitumumab in combination with gefitinib or afatinib also improved efficacy against EGFR-del19/C797S and brigatinib-resistant triple mutants compared to single treatment with gefitinib or afatinib (Fig. 4a).

However, brigatinib-resistant quadruple mutants conferred marked resistance to any of the currently approved EGFR-TKIs (Fig. 4b). Therefore, to identify novel therapeutic drugs to overcome these ultimate brigatinib-resistant compound mutants, we further examined drugs, including the fourth-generation EGFR inhibitor BI-4020, HER2 inhibitors TAK165, TAK285, and BDTX-189, and the EGFR inhibitor AZD3759.

As a result, BI-4020 effectively inhibited almost all types of brigatinib-resistant triple mutants with IC50 values < 10 nM and the combination of BI-4020 with panitumumab decreased the IC_50_ values by ~3–10 folds compared with a single treatment of BI-4020, and mostly <1 nM (Fig. 4a). We then validated the inhibitory effects of gefitinib, afatinib, osimertinib, brigatinib, and BI-4020 on the EGFR-del19/C797S/L718M and EGFR-del19/C797S/L718Q mutants using immunoblot analysis (Supplementary Fig. 2a, b). The results showed that both the EGFR-del19/C797S/L718M and EGFR-del19/C797S/L718Q mutants had decreased phosphorylation levels at ~10 nM concentrations of gefitinib, afatinib, and BI-4020, although at >100 nM concentrations for brigatinib (Supplementary Fig. 2a, S2b).

Furthermore, the brigatinib-resistant quadruple mutants were highly sensitive to BI-4020 or BI-4020 in combination with panitumumab (Fig. 4b). Western blotting of BI-4020 single treatment showed that almost all mutants, except for the G796N compound mutant, showed a marked decrease in phosphorylation level at 10 nM, especially for the EGFR-activating mutation/T790M/C797S/L718M mutant, which showed a reduction in phosphorylation level at 1 nM (Fig. 4c). BI-4020 with panitumumab further induced a reduction in the phosphorylation level of EGFR and downstream signals in the Ba/F3-del19/T790M/C797S/L718M mutants (Supplementary Fig. 3a). The inhibition of phospho-EGFR by BI-4020 was maintained at least until 24 h (Supplementary Fig. 3b).

In contrast, the HER2 inhibitors TAK165, TAK285, and BDTX-189 were not effective against the brigatinib-resistant triple mutants (Supplementary Fig. 4a–c). TAK165 inhibited the growth of all Ba/F3 EGFR mutant cells at ~30–100 nM, whereas TAK285 did not inhibit any type of EGFR mutant-expressing cells (Supplementary Fig. 4a, b). BDTX-189 can only inhibit the growth of Ba/F3-del19 cells, but not other C797S-harboring cells, probably due to the loss of the covalent binding residue, since BDTX-189 has a Michael acceptor (Supplementary Fig. 4c). On the other hand, AZD3759 effectively inhibited Ba/F3-del19 and most of the C797S harboring compound mutants, such as del19/C797S/L718X, but it was not effective against T790M harboring EGFR mutants (Supplementary Fig. 4d).

### Efficacy of BI-4020 to EGFR mutant non-small-cell lung cancer cells

Next, we examined the drug sensitivity of the PC9 cells expressing various EGFR mutations. The addition of each L718M/Q/F mutation to the triple mutant resulted in an increase in the IC_50_ value of brigatinib compared to that of the del19/T790M/C797S mutant, and these mutants also showed resistance to all clinically available EGFR-TKIs (Fig. 4d). BI-4020 showed lower IC_50_ values than conventional EGFR inhibitors against all EGFR mutants used in this study. In particular, the IC_50_ value of BI-4020 in PC9 cells expressing the EGFR-del19/T790M/C797S/L718M mutant was <10 nM, suggesting that the L718M mutant could be overcome by BI-4020. Furthermore, the IC_50_ values of del19, del19/C797S, and del19/T790M/C797S mutants were <50 nM, suggesting that BI-4020 has stronger inhibitory effects than conventional EGFR inhibitors (Fig. 4d).

To examine the selectivity of BI-4020 for EGFR mutants, the sensitivity was examined using wild-type EGFR amplified and highly expressing A431 cells or KRAS mutation-positive A549 cells (Fig. 4e). In BI-4020 monotherapy and panitumumab combination treatment, del19, del19/C797S, del19/T790M/C797S, and del19/T790M/C797S/L718M mutants showed higher selectivity than wild-type EGFR-expressing A431 cells, suggesting that osimertinib-resistant mutant and brigatinib-resistant compound mutants can be overcome by BI-4020 (Fig. 4e). The result of KRAS mutation-positive A549 cells treated with BI-4020 suggested that driver mutation-positive cancers other than EGFR would be insensitive to BI-4020 (Fig. 4e).

**Fig. 5:**
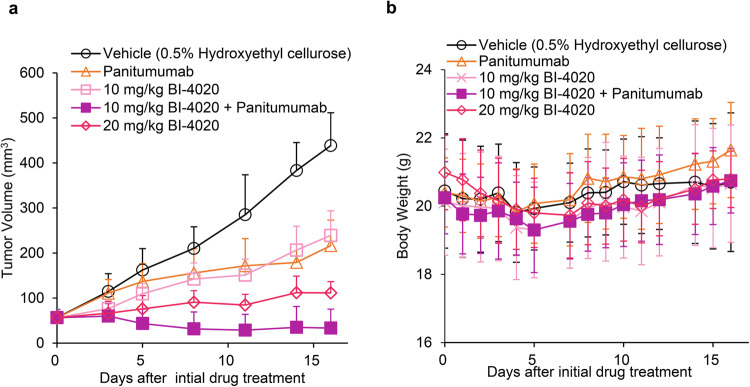
BI-4020 with anti-EGFR antibody overcomes brigatinib-resistant compound mutants in vivo. **a** PC9-del19/T790M/C797S/L718M mutant cells were subcutaneously injected into nude mice, and treated with the indicated doses of panitumumab, BI-4020, or BI-4020 and panitumumab combination for 16 days. Average tumor volume was plotted as mean ± SD (*n* = 6). **b** Average body weight was presented as mean ± SD.

Notably, TAK165 inhibited the growth of all EGFR mutant-expressing PC9 cells at ~30–100 nM, suggesting that TAK165 might induce cell death in PC9 cells by inhibiting different targets such as electron transport chain complex I (Supplementary Fig. 5a)^28^. Similar to the results observed in the Ba/F3 models, TAK285 did not show efficacy, and BDTX-189 was effective only when the C797S mutation did not exist (Supplementary Fig. 5b, c). AZD3759 was effective for the EGFR-del19 and EGFR-del19/C797S mutants, but not for the T790M-containing mutants (Supplementary Fig. 5d). Western blot analysis using PC9-del19/T790M/C797S/L718M cells showed a significant attenuation of phospho-EGFR at 10 nM BI-4020 treatment. Phosphorylation of the downstream signals of EGFR was also reduced, with a complete loss of phospho-ERK and phospho-S6 in a dose-dependent manner. (Fig. 4f and Supplementary Fig. 6a). The inhibition of phospho-EGFR by BI-4020 treatment was observed for at least up to 48 h (Supplementary Fig. 6b). When panitumumab was treated with BI-4020, further attenuation of phosphorylation of EGFR and its downstream molecules was observed in PC9-del19/T790M/C797S/L718M and -del19/T790M/C797S/L718Q mutant cells (Fig. 4f, Supplementary Fig. 7). In addition, BI-4020 plus panitumumab combination therapy induced significant tumor shrinkage in vivo without any change in body weight (Fig. 5a and b), whereas osimertinib failed to inhibit the growth of PC9-del19/T790M/C797S/L718M xenograft tumors (Supplementary Fig. 8).

These results suggest that gefitinib, afatinib, and BI-4020 are effective against brigatinib-resistant triple mutants without T790M, such as the activating mutation/C797S/L718X. Moreover, BI-4020 and BI-4020 combined with panitumumab were effective against brigatinib-resistant quadruple mutants. It was also suggested that BI-4020 is effective in the treatment of pre-existing del19, del19/C797S, and del19/C797S/T790M mutants compared to wild-type EGFR.

### BI-4020 is not effective against EGFR minor mutations

**Fig. 6:**
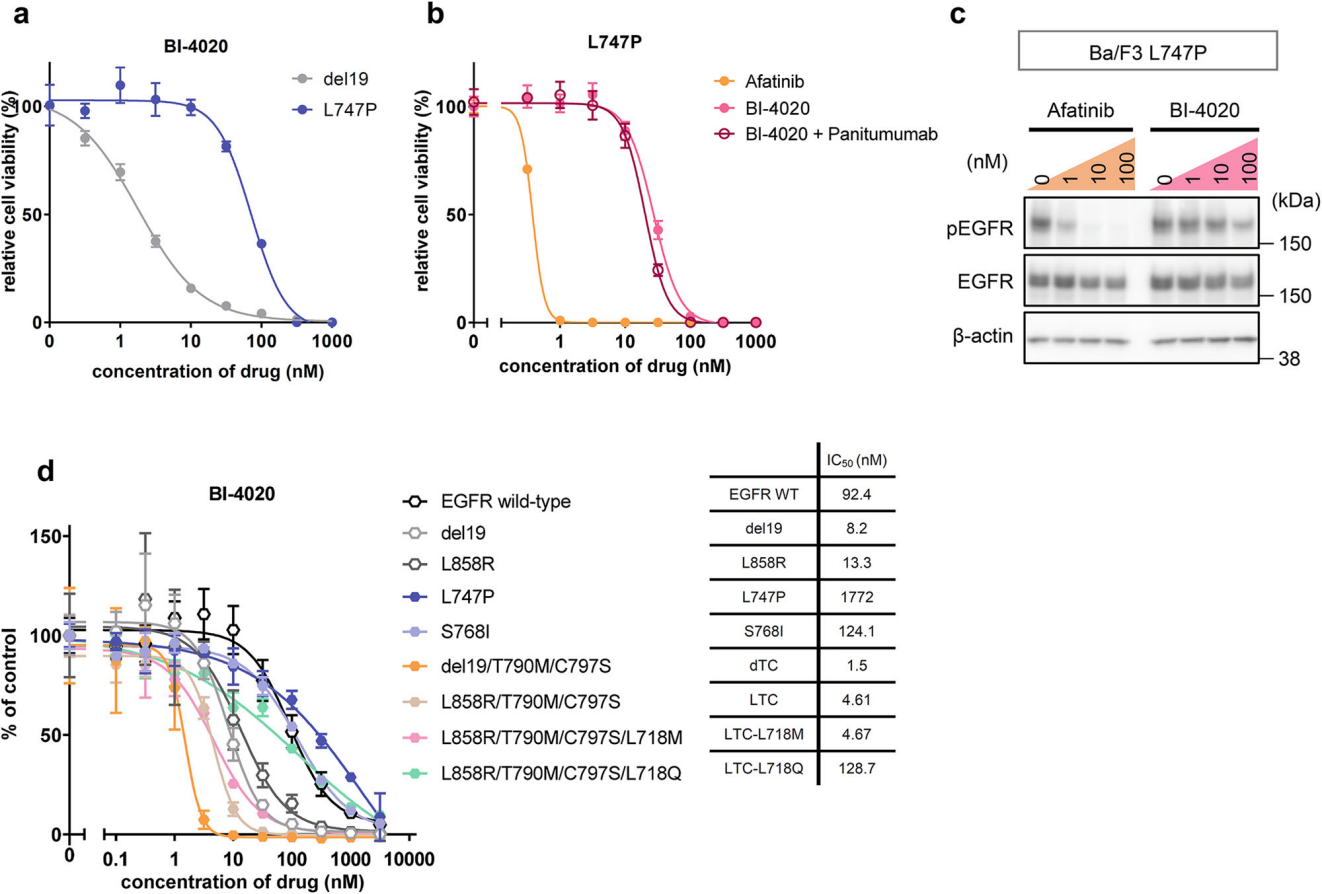
BI-4020 is not effective for EGFR minor mutants. **a** Ba/F3-del19 and Ba/F3-L747P cells were treated with serially diluted BI-4020 for 72 h. The viability was assessed by using the CellTiter-Glo assay. Experiments were repeated three times independently. **b** Growth inhibition was assessed by CellTiter-Glo assay for Ba/F3-L747P cells. Cells were treated with serially diluted afatinib, BI-4020, and BI-4020 and in combination with panitumumab (10 µg/ml) for 72 h. **c** Western blotting analysis for Ba/F3-L747P mutant. The indicated concentration of drugs was treated for 3 h. Collected cell lysates were analyzed by immunoblot with the indicated antibodies. Uncropped immunoblot images are presented in Supplementary Fig. 10. **d** The evaluation of the inhibitory activity of BI-4020 in the in vitro kinase assay using the ADP-Glo assay. The indicated purified EGFR mutants were incubated with 100 µM of ATP, substrates, and serially diluted inhibitors. After 1 h of incubation at room temperature, the synthesized ADP level was measured by the ADP-Glo assay kit.

Our results suggest that BI-4020 may be able to overcome a wide variety of EGFR-C797S compound mutants, including brigatinib-resistant quadruple mutants that are resistant to all existing EGFR inhibitors. In addition, BI-4020 effectively inhibited the growth of parental EGFR mutant cells harboring only the major activating mutations (del19 and L858R). When considering the first-line use of BI-4020, it would be better to evaluate its efficacy of BI-4020 not only for the major EGFR-activating mutants but also for a minor mutant, such as the L747P mutant that has been diagnosed as del19 by a routine PCR-based diagnostic test and previously reported from our laboratory^29^. In contrast to the major EGFR-activating mutants, BI-4020 was not effective against the L747P mutant (Fig. 6a). Compared to afatinib, BI-4020 did not show efficacy in the L747P mutant (Fig. 6b, c). Interestingly, there was no additive effect on the EGFR-L747P mutant even when combined with the anti-EGFR antibody panitumumab (Fig. 6b). We also performed an in vitro kinase assay and found that BI-4020 was ineffective against the S768I EGFR minor mutant (Fig. 6d).

### Efficacy of BLU-945 to EGFR mutant Ba/F3 cells

**Fig. 7:**
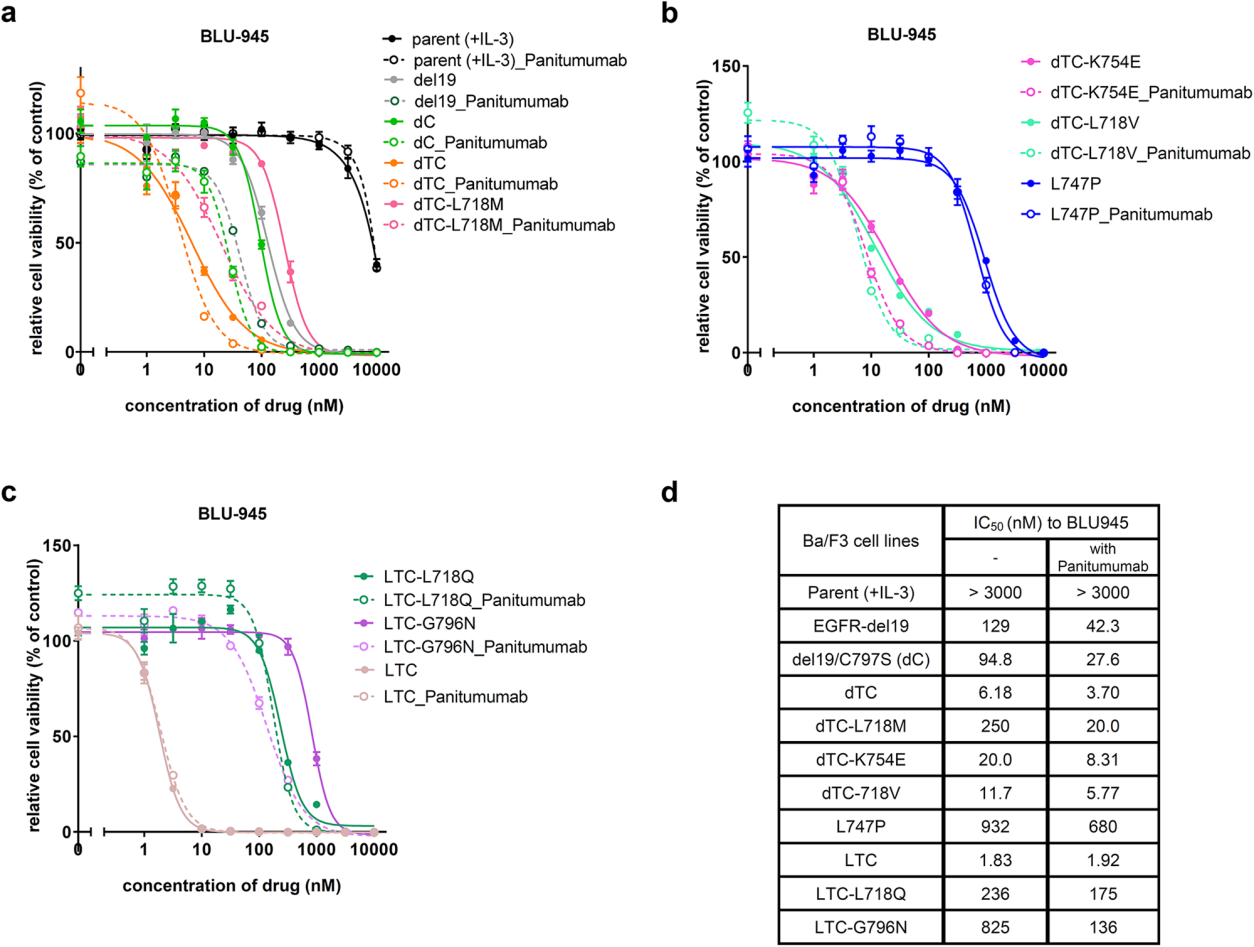
Activity of BLU-954 to EGFR mutants expressing Ba/F3 cells. **a**–**c** The selectivity of BLU-954 and combination treatment with panitumumab (10 µg/ml) using the indicated Ba/F3 parental or EGFR mutants expressing Ba/F3 cells. Cells were treated with each drug for 72 h. **d** IC_50_ values were calculated from the cell viability assay data in (**a**–**c**). Results are presented as mean ± SD.

Next, we investigated the sensitivity of BLU-945, a fourth-generation EGFR-TKI currently in clinical evaluation, to the Ba/F3 cells expressing different EGFR mutations. As previously reported^25^, BLU-945 showed lower IC_50_ values against osimertinib-resistant triple mutants (L858R/T790M/C797S or del19/T790M/C797S) with an IC50 of < 10 nM. In contrast, the IC50 of BLU-945 against Ba/F3 cells expressing the EGFR del19/T790M/C797S/L718M mutant was approximately 250 nM, indicating that the L718M mutant could not be overcome by BLU-945 monotherapy. However, combination treatment of BLU-945 with panitumumab, an anti-EGFR antibody, showed a significant reduction in IC_50_ (around 20 nM) against the EGFR L718M quadruple mutant Ba/F3 cells. BLU-945 was also highly active against del19/T790M/C797S/L718V or del19/T790M/C797S/K754E mutants, but not against L858R/T790M/C797S/L718Q or L858R/T790M/C797S/G796N resistant mutations. Of note, BLU-945 was less potent against del19 single, del19/C797S double mutants and inactive against EGFR-L747P activating mutation. And addition of EGFR antibody to BLU-945 increased the sensitivity to del19 or del19/C797S cells but not to L747P mutant at all (Fig. 7). These results suggest that BLU-945 has strong inhibitory effects on several quadruple resistant mutants, but the activity to each resistant mutant or activating mutations is very different among the mutation position and residues.

### Analysis of binding mode of BI-4020 to EGFR mutants through MD simulation

**Fig. 8:**
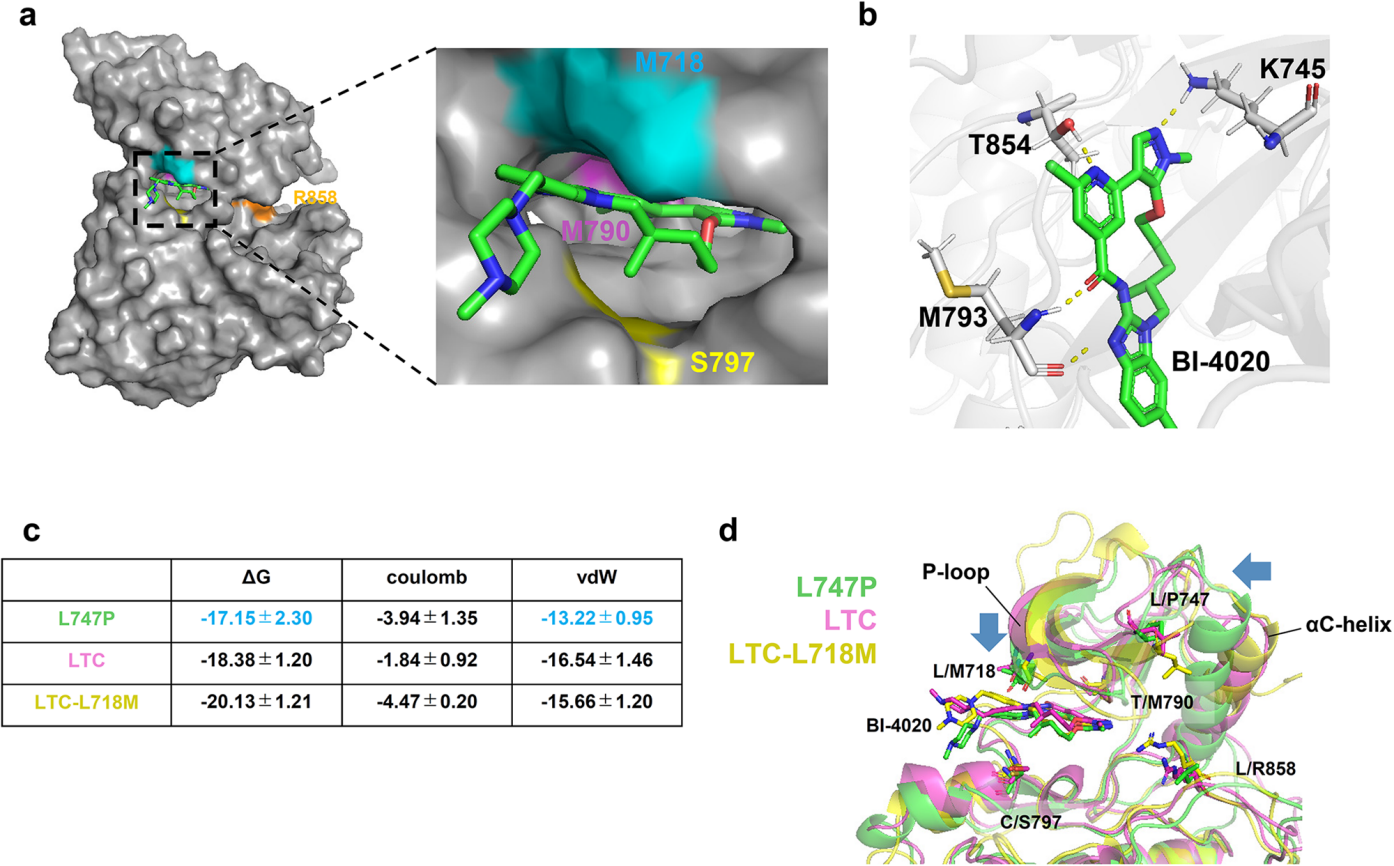
Structural model of EGFR–BI4020 interactions. **a** The binding mode of BI-4020 for the EGFR-L858R/T790M/C797S/L718M mutant. EGFR was depicted by a surface model (M718, cyan; M790, red; S797, yellow; R858, orange; others, gray), and BI-4020 was depicted by sticks (C, green; N, blue; and O, red). An enlarged view of the ATP-binding pocket is shown in the right panel. **b** Hydrogen bonds between the EGFR-L858R/T790M/C797S/L718M mutant and BI-4020. The protein backbone is represented by a ribbon diagram, and the side chains (K745, M793, and T854) and BI-4020 are depicted as sticks. Hydrogen bonds are shown as dashed yellow lines. **c** Binding free energies (Δ*G*) of BI-4020 toward the L747P, L858R/T790M/C797S, and L858R/T790M/C797S/L718M mutants. Electrostatic (Coulomb) and van der Waals (vdW) contributions to the ΔG values are also indicated. The binding affinity for the L747P mutant is significantly lower than that for the L858R/T790M/C797S or L858R/T790M/C797S/L718M mutant owing to the loss of vdW interactions. **d** Structural comparison of the L747P, L858R/T790M/C797S, and L858R/T790M/C797S/L718M mutants. Orientational changes in the phosphate-binding loop (P-loop) and αC-helix upon the L747P mutation are indicated by blue arrows. In **a**–**d**, the energetically stable structure of the EGFR–BI-4020 complex was extracted from five independent 100 ns molecular dynamic simulations.

To understand the binding mode of BI-4020 to EGFR mutants (L858R/T790M/C797S, L858R/T790M/C797S/L718M, and L747P), we performed MD simulations of these EGFR mutant kinases, whose initial structures were computationally modeled based on the crystal structure of the wild-type EGFR kinase complexed with BI-4020 (PDB 7KXZ). BI-4020 fitted into the ATP-binding pocket of EGFR-L858R/T790M/C797S/L718M and formed hydrogen bonds between the K745, T854, and M793 residues (Fig. 8a, b). To further understand the binding affinity of BI-4020 to EGFR mutants, the binding Free Energy (Δ*G*) values of BI-4020 toward EGFR-L747P, -L858R/T790M/C797S, or -L858R/T790M/C797S/L718M were calculated in silico by Massively Parallel Computation of Absolute binding Free Energy with well-equilibrated states (MP-CAFEE)^30^. Consistent with the cell viability assay and immunoblot assay using Ba/F3 cells expressing each EGFR mutant, the L747P mutant showed higher Δ*G* values (−17.15 kcal/ml) compared with the L858R/T790M/C797S (−18.38 kcal/mol) or L858R/T790M/C797S/L718M (−20.13 kcal/mol) mutants (Fig. 8c). The decrease in binding affinities of L747P−BI-4020 compared with L858R/T790M/C797S− or L858R/T790M/C797S/L718M−BI-4020 was caused by a decrease in van der Waals interactions (Fig. 8c). As shown in Fig. 8d and Supplementary Fig. 9, the L747P mutation seems to promote the active conformation by changing the orientation of the αC-helix and affecting the binding affinities of EGFR-TKIs by restricting the conformational flexibility of the P-loop, which forms part of the ATP-binding pocket, as we recently reported^29^.

## Discussion

In this study, we showed that brigatinib with any of the anti-EGFR antibodies was effective against osimertinib-resistant mutants in vitro and in vivo. In Fig. 1b–d, since we reduced the dose of brigatinib compared to that used in our previous study^19^, the dose used in this study is speculated to achieve the concentration in the human body at the approved dose of brigatinib for ALK-rearranged cancer. As the brigatinib-based combination therapies induced marked tumor growth suppression in mice for at least over 1 month, we expect that the combination therapies might be effective in humans.

Our crystal structure analysis revealed the binding mode of brigatinib to the EGFR-T790M/C797S mutant, and through ENU mutagenesis screening, brigatinib-resistant compound mutations were identified, such as L718M/V/Q, K754E, and G796N mutations, which were reported as osimertinib-resistant mutations in clinical settings. Although brigatinib-resistant triple mutants (activating mutation/C797S/+ newly identified mutations) could be overcome by gefitinib or afatinib, brigatinib-resistant quadruple mutants were resistant to all the conventional EGFR inhibitors. Our results revealed that the fourth-generation EGFR inhibitor BI-4020 could overcome these quadruple mutants. The efficacy of BI-4020 was also evaluated in vivo, and a significant anti-tumor effect against the EGFR-del19/T790M/C797S/L718M quadruple mutant was confirmed using BI-4020 with an anti-EGFR antibody combination without any apparent side effects.

Crystal structure analysis of brigatinib complexed with EGFR-T790M/C797S mutant kinase showed that the hydrogen bonds between M793 of EGFR and brigatinib play an important role in their binding, and L718, L792, and G796 were in close proximity to EGFR-bound brigatinib. Notably, this binding mode was almost identical to the predicted binding mode as per our previous computational MD simulation^19^. Therefore, we expect that the predicted binding mode of BI-4020 to EGFR quadruple mutants evaluated through an MD simulation in this study would correctly reflect the actual binding mode. Mutations in L718 or G796 residues, such as L718Q or G796R/S/D, were also found to induce osimertinib resistance^17^.

On the other hand, it was inferred from crystal structure analysis (PDB:7kxz) that BI-4020 forms hydrogen bonds with four sites (K745, M793, E804, and T854). BI-4020 was ineffective against L747P because L747P maintains a very stable salt bridge between K745 and E762 to form a stable active conformation of EGFR, which may prevent the formation of hydrogen bonds between K745 and BI-4020. The S768I mutant also showed limited efficacy against BI-4020, based on the findings of the in vitro kinase assay. In 2021, Robichaux et al. reported the structure of EGFR mutants and their sensitivity to various EGFR inhibitors and found that L747P and S768I are classified in the P-loop and αC-helix compressing group^4^. These structural changes may affect the binding of BI-4020. It is important to examine the efficacy of BI-4020 against other EGFR minor mutants in future studies.

BI-4020, a fourth-generation EGFR inhibitor, was developed to overcome the EGFR-activating mutation/T790M/C797S triple mutation. Unlike conventional EGFR inhibitors, it is a macrocyclic structure inhibitor with a reported binding selectivity of approximately 150-fold for EGFR mutants^27^, which may lead to new therapeutic strategies for mutants that are difficult to overcome. Our study showed that BI-4020 had higher inhibitory activity than the other EGFR inhibitors and higher selectivity for the L718M mutation than for the wild-type EGFR. The in vitro kinase assay also showed that L858R/T790M/C797S had a lower IC50 value against BI-4020 than L858R mutation alone. This may be because the L858R mutant does not affect the overall structure of EGFR^4,31^. Although the selectivity for EGFR mutants is very high compared to wild-type EGFR, careful evaluation of adverse events will be needed in future studies.

In addition to BI-4020, multiple fourth-generation EGFR inhibitors have been under development. Among them, BLU-945, which is currently undergoing clinical trials, is an ATP competitive inhibitor similar to BI-4020 and has been developed to overcome the EGFR-activating mutation/T790M/C797S. According to the previous report^25^, BLU-945 is predicted to form hydrogen bonds with K745, Q791, M793, G796, K716, and K728 (PDB ID:8D73, 8D76); given the predicted hydrogen bond with G796, overcoming the EGFR-activating mutation/T790M/C797S/G796 mutant might be difficult by BLU-945. Moreover, BLU-945 may have difficulty overcoming L747P because it is expected to form a hydrogen bond with K745, similar to BI-4020. In addition, JBJ-09-063 and EAI045, fourth-generation allosteric EGFR inhibitors, bind to the allosteric pocket in the C-helix-out conformation of EGFR^32^. Unlike BI-4020 and BLU-945, these allosteric inhibitors have been reported to be effective against L858R and L858R/T790M mutants, but not against del19 mutants^32,33^. In addition, these allosteric inhibitors have been reported to have relatively low antitumor activity as single agents. JBJ-09-063 in combination with osimertinib and EAI045 in combination with an anti-EGFR antibody are known to enhance their activity^32,33^. In our study, BI-4020 as a single treatment was found to be particularly effective against the L718M compound mutants, and the combination of BI-4020 and panitumumab showed further growth inhibition against any quadruple mutants and remarkable in vivo anti-tumor effects against the L718M compound mutation. The reason for this is assumed from a previous study^19^ that the combination of an anti-EGFR antibody inhibits both the dimerization and activation of EGFR by inducing its internalization. However, since the IC50 values were approximately 3–10 fold lower than that of BI-4020 single treatment, anti-EGFR antibody binding to the extracellular domain might induce conformational changes in both EGFR-del19 and -L858R mutants. In our previous study^29^, we showed that the EGFR L747P mutation, which has an inflexible C-helix-in conformation, and the addition of anti-EGFR antibody did not change the IC50 values of any EGFR inhibitor. Further studies are needed to determine how EGFR antibody binding affects the conformation of EGFR and increases the sensitivity of EGFR inhibitors. In addition, the development of new macrocyclic structure inhibitors, such as analogs of BI-4020 or other structurally distinct next-generation EGFR inhibitors, may be needed to overcome the various emerging mutants and to target minor EGFR-activating mutants.

This study had a few limitations. First, ENU mutagenesis is biased in that it often induces A → G mutations, whereas G → C mutations are less likely to occur, as reported previously^34^. Therefore, other brigatinib-resistant mutations may emerge in the clinical setting. Second, a comparison with other fourth-generation EGFR inhibitors is required. It is necessary to confirm whether the results obtained with BI-4020 are better than those obtained with other fourth-generation EGFR inhibitors. In addition, although this study focused on mutations occurring within the tyrosine kinase domain of EGFR, the expected frequency of occurrence of these compound mutations would be low. Therefore, identification of resistance mechanisms other than compound mutations and strategies methods to overcome the other mechanisms such as other mutations and the activation of bypass signaling should also be investigated.

In conclusion, our findings suggest that brigatinib-resistant mutants can be overcome by BI-4020, a fourth-generation EGFR inhibitor, as a single treatment or in combination with an anti-EGFR antibody, although further evaluation of the safety and efficacy of BI-4020 in vivo is needed to consider its clinical application. The development of macrocyclic inhibitors, such as BI-4020 derivative and other fourth-generation EGFR inhibitors, may overcome resistant mutants that may emerge in the future.

## Methods

### Cell lines and reagents

Ba/F3 immortalized murine bone marrow-derived pro-B cells were cultured in Dulbecco’s minimal essential medium (DMEM) low glucose (Wako) supplemented with 10% fetal bovine serum (FBS) and 250 µg/ml kanamycin (Meiji Seika Pharma) (D-10) with or without 0.5 ng/ml of interleukin (IL)-3 (Invitrogen). Human epidermoid carcinoma cell line A431 and human adenocarcinoma cell line A549 were cultured in D-10. PC9, a human cancer cell line derived from lung adenocarcinoma cells, was cultured in RPMI 1640 (Wako) supplemented with 10% FBS and kanamycin (R-10). 293FT human embryonic kidney cells were cultured in DMEM high glucose (Wako) supplemented with 10% FBS. Detailed information about the reagents used in this study is described in Supplementary Table 2.

### Establishment of oncogene-expressing cell lines

For lentivirus production, pLenti6.3 vectors containing each EGFR mutant were transfected into 293FT cells using packaging plasmids (ViraPower, Thermo Fisher Scientific). Ba/F3 cells were infected using lentivirus-containing medium supplemented with 8 µg/ml polybrene for 24 h. Then, the infected cells were selected by culturing for 1 week with 7 µg/ml blasticidin. After selection, each cell line was cultured in D-10 without IL-3.

### ENU mutagenesis screening

Ba/F3-EGFR-del19/C797S, Ba/F3-EGFR-del19, and L858R/T790M/C797S cells (1 × 10^6^ cells/ml) were exposed to 100 µg/ml N-ethyl-N-nitrosourea (ENU) (Sigma) for 24 h. After ENU treatment, the cells were washed with PBS and cultured on D-10 for 24 h. The cells were seeded at a density of 1 × 10^5^ cells/well in 96-well plates for 24 h and cultured with brigatinib (300, 600, 800, or 1000 nM), a combination of brigatinib (300, 600, 800, or 1000 nM), 10 µg/ml panitumumab, or gefitinib (300, 600, or 1000 nM) for 3 weeks. Drug-resistant cells were picked and expanded. DNA extracts were prepared from these clones by lysing them with proteinase K buffer, and the entire kinase domain of EGFR was amplified using KOD plus neo (TOYOBO). The PCR products were purified using a gel purification kit (GE Healthcare) and analyzed using standard Sanger sequencing.

### Cell viability assay

Ba/F3 cells (2000 or 3000 cells/well) were seeded in 96-well black plates and cultured in a medium containing serially diluted drugs for 72 h. PC9 cells (1500 cells/well) were seeded in 96-well black plates for 24 h and treated with serially diluted drugs for 72 h.

After drug treatment, cell viability was measured using the CellTiter-Glo assay (Promega) and luminescence was measured using TriStar LB941 (Berthold Technologies). The IC50 values were assessed using GraphPad Prism version 7.0.4 (GraphPad software). The experiments were independently repeated thrice.

### Antibodies and immunoblotting

After drug treatment, cells were washed with cold PBS and lysed using SDS lysis buffer (100 mM Tris, 1% SDS, 10% glycerol). Lysates were boiled at 100 °C for 5 min. Protein concentrations were measured using BCA Protein Assay Reagent (Thermo Fisher Scientific) following the manufacturer’s protocol. Equal amounts of protein were separated by SDS–PAGE and transferred to polyvinylidene difluoride membranes. Antibodies against phospho-EGFR (Tyr 1068; GTX132810, GeneTex, 1:2000, or Tyr1173; Cell Signaling Technology, #4407, 1:1000), total EGFR (Cell Signaling Technology, #4267, 1:2000), phospho-Akt (Ser473; Cell Signaling Technology, #4060, 1:1000), total Akt (Cell Signaling Technology, #4691, 1:5000), phospho-ERK (Thr202/Tyr204; Cell Signaling Technology, #9101, 1:5000), total ERK1/2 (Cell Signaling Technology, #9102, 1:2000), phospho-S6 (Ser240/244; Cell Signaling Technology, #5364, 1:10,000), total S6 (Cell Signaling Technology, #2217, 1:2000), and β-actin (Sigma-Aldrich, A5228, 1:10,000 or Sigma-Aldrich, clone; AC-15, 1:10,000) were used. Uncropped Images are presented in Supplementary Fig. 10.

### In vitro kinase assay

Recombinant proteins of the kinase domain of wild-type EGFR and each EGFR mutant were synthesized by Carna Biosciences^35^. The appropriate amount of target proteins was calculated with the ADP-Glo assay (Promega) according to the manufacturer’s protocol and incubated in 96-well half-area white plates with serially diluted inhibitor over a 10-dose range from 0.1 to 3160 nM for 10 min at room temperature. ATP (100 µM) was mixed with 200 µg/ml substrate, added to the kinase protein-inhibitor mixture, and then incubated for 60 min at room temperature. After the kinase reaction, an equal volume of ADP-Glo Reagent was added to terminate the reaction, and the remaining ATP was depleted. The Kinase Detection Reagent was added to convert ADP to ATP and to allow the newly synthesized ATP to be measured by luciferase assay. Luminescence was measured using TriStar LB941.

### In vivo evaluation of brigatinib or BI-4020 with anti-EGFR antibody

All mouse studies were performed under the Institutional Animal Care and Use Committee–approved animal protocols, according to the institutional guidelines. PC9-del19/T790M/C797S (PC9/Triple) (4 × 10^6^) or PC9-del19/C797S (4 × 10^6^) cells were suspended in 100 µl of HBSS and subcutaneously implanted into BALB/c nu/nu mice (Charles River). Tumor growth was monitored twice weekly by bilateral caliper measurement, and tumor volume was calculated as 0.5 × length × width × width (mm^3^). When the average tumor volume reached approximately 100–200 mm^3^, the mice were randomized into vehicle and treatment groups according to the tumor size. Mice were treated once daily by oral gavage for the indicated period. Relative tumor volume was calculated by dividing the tumor volume on day 0. The body weights of the mice were measured twice weekly. The mice were euthanized within several days when the tumor size exceeded 1000 mm^3^ by cervical dislocation. The Mann–Whitney *U* test was used for statistical analysis of the mouse experiments.

### Crystal structure analysis of EGFR-T790M/C797S with brigatinib

The human EGFR (residues 696-1022) T790M/C797S mutant was expressed and purified using *Spodoptera frugiperda* (Sf9) cells as previously reported^36^. Attempts to co-crystallize EGFR-T790M/C797S and brigatinib yielded only tiny crystals, whereas crystallization of EGFR-T790M/C797S alone (apo-form) succeeded in obtaining diffraction-quality crystals with a resolution of 2.4 Å. The best crystals of the apo-form EGFR-T790M/C797S grew in 0.2 M sodium tartrate dibasic dihydrate and 22% PEG3350 at 20°C using the hanging-drop vapor diffusion method by mixing 1 µl of protein solution (15 mg/ml) and 1 µl of the reservoir solution. The apo-form crystals were then soaked overnight in 10 µM brigatinib and flash-cooled in liquid nitrogen in a reservoir solution containing 15% glycerol. X-ray data were collected at 100 K at a wavelength of 1.0 Å the SPring-8 beamline BL26B2 (Harima, Japan). The data were processed using the XDS program^37^ and the structure was determined using the molecular replacement method with the Phaser program^38^. The crystal structure of EGFR-T790M/C797S in complex with Go6976 (PDB ID:5XGN) was used as a search model. The model was built and refined iteratively using the Coot^39^ and Phenix^40^. The quality of the model was evaluated using PROCHECK software^41^. Molecular graphics were generated using the PyMOL software^42^.

### Molecular dynamics (MD) simulation of wild-type EGFR or its mutants in complex with BI-4020

The initial structure of wild-type EGFR in complex with BI-4020 was obtained from the Protein Data Bank (PDB ID:7KXZ). The structures of disordered loops and flexible side chains were modeled using the Structure Preparation module in the Molecular Operating Environment (MOE) program v. 2016.08^43^. The N- and C-termini were capped with acetyl and N-methyl groups. The titratable residues remained in their dominant protonation state at pH 7.0. The L718M, L747P, T790M, C797S, and L858R mutations were introduced into the structure of wild-type EGFR using the Structure Preparation module in MOE. BI-4020 was protonated to form an ionized state in the solution (net charge of +2).

All MD simulations were performed using the GROMACS 2021 program^44^. As previously reported^19^, computational systems of the EGFR−BI-4020 complexes were prepared, and their MD simulations were performed. For each EGFR mutant, five sets of 100 ns production runs and three sets of 1 μs production runs were executed with different velocities. Three sets of 20 ns production runs were carried out for the solvated BI-4020 system. The binding free energy (Δ*G*) of BI-4020 toward EGFR-L747P, L858R/T790M/C797S, or L858R/T790M/C797S/L718M was calculated using MP-CAFEE, which constitutes one of the biochemical free energy perturbation methods^30^. The Δ*G* value for each EGFR mutant was calculated as previously described^45^.

### Data and statistical analysis

Data are presented as the mean ± SD unless otherwise specified. Pairwise comparisons between groups were performed using paired or unpaired Student’s *t*-tests, as appropriate. Significant probability (*P*) values are indicated as ***P < 0.001, **P < 0.01, and *P < 0.05.

### Reporting summary

Further information on research design is available in the Nature Research Reporting Summary linked to this article.

### Supplementary information

Supplementary Material

Reporting Summary

## Data Availability

All reasonable requests for resources and reagents should be directed and will be fulfilled by the Lead Contact author (Ryohei Katayama: ryohei.katayama@jfcr.or.jp). The materials and data will be made available upon request after the completion of a material transfer agreement. The authors declare that all data supporting the findings of this study are available within the paper or deposited publicly (Protein Data Bank: 8H7X).
