# Supplementary material for: A macrocyclic kinase inhibitor overcomes triple resistant mutations in EGFR-positive lung cancer
Source: NPJ Precis Oncol. 2024 Feb 23;8:46. doi: 10.1038/s41698-024-00542-9 (PMC10891166; doi:10.1038/s41698-024-00542-9)
Supplement: Supplementary file 1 — Supplementary Material [file 41698_2024_542_MOESM1_ESM.pdf]

Supplementary Figure 1

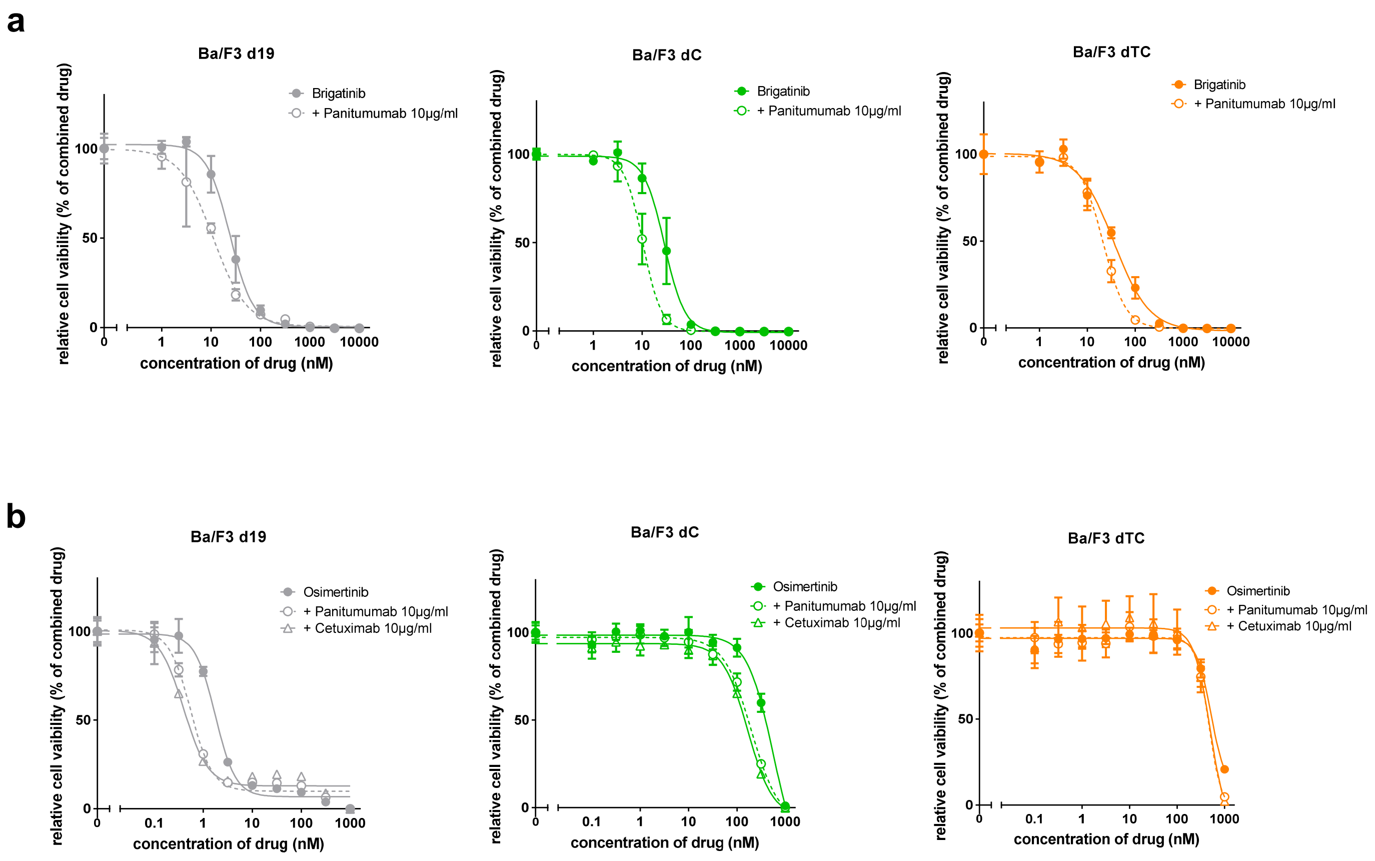

Supplementary Figure 1. Cell viability assay of Ba/F3 EGFR-mutants cells treated with osimertinib with EGFR antibodies.

(a, b) Each mutant cells were treated with serially diluted Brigatinib +/- Panitumumab (10 µg/mL) (a), or serially diluted Osimertinib +/- Cetuximab (10 µg/mL) or Panitumumab (10 µg/mL) for 72 h. The viability was assessed by using a CellTiter-Glo assay.

Supplementary Figure 2

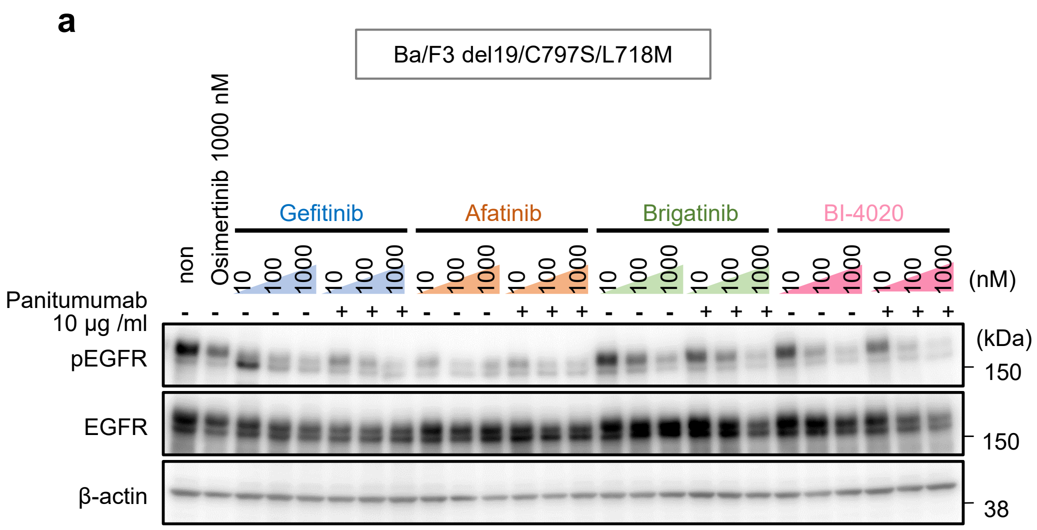

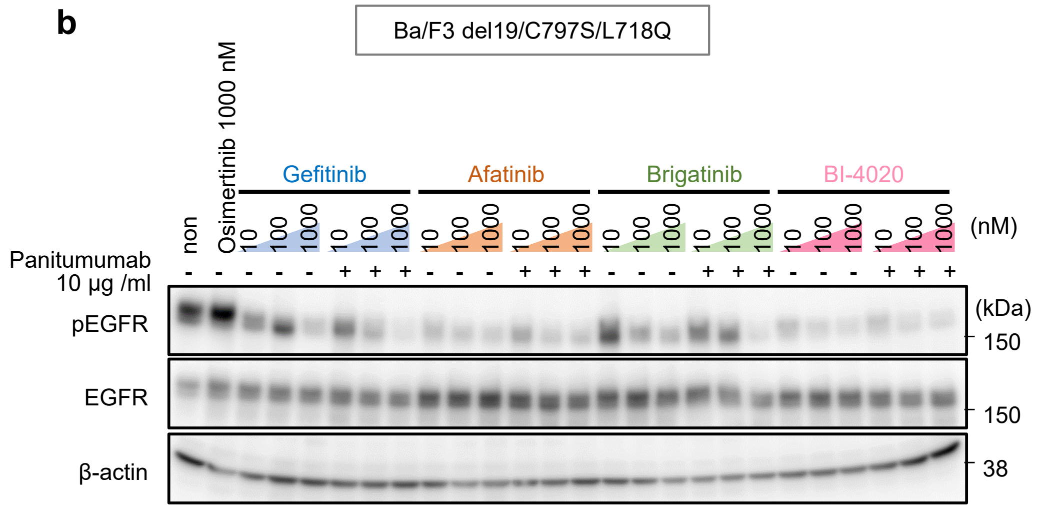

Supplementary Figure 2. Western blotting analysis of Ba/F3 ENU clone cells
Ba/F3-EGFR-del19/C797S/L718M (a) and Ba/F3-EGFR-del19/C797S/L718Q cells (b) were treated with each concentration of drugs for 6 h. Collected cell lysates were analyzed by immunoblot with the indicated antibodies.

Supplementary Figure 3

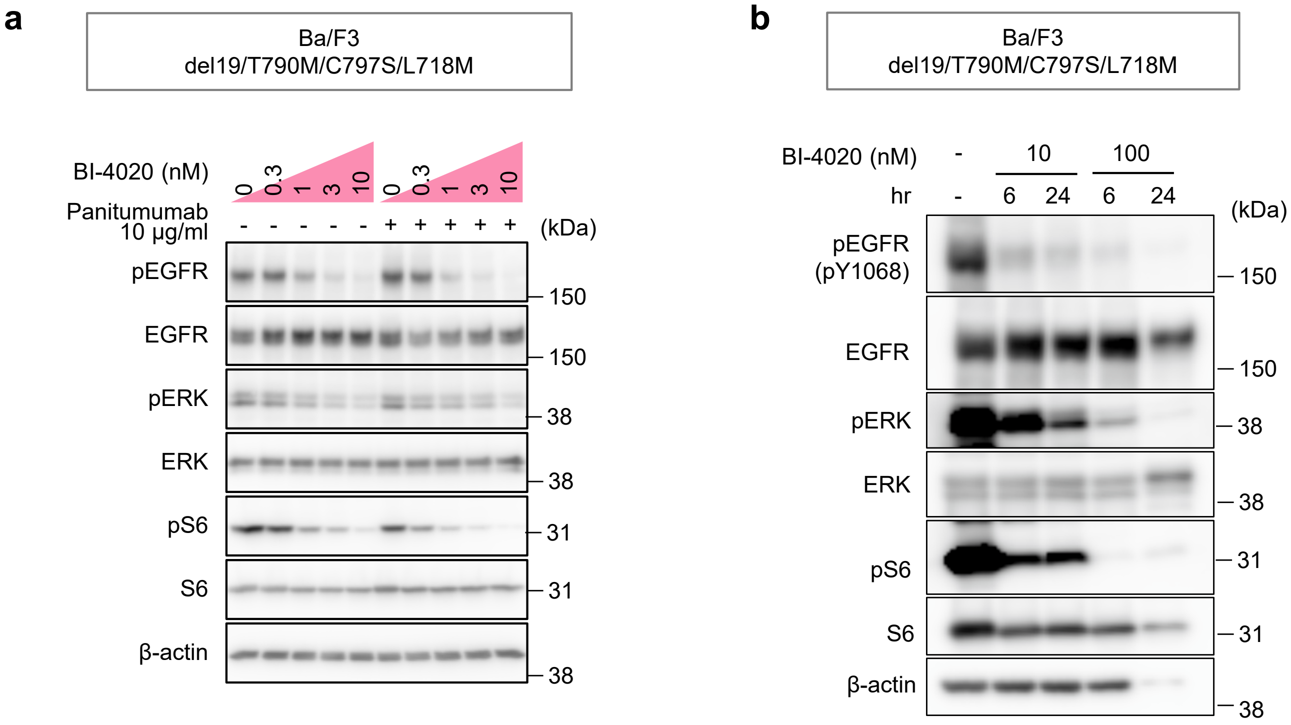

Supplementary Figure 3. Western blotting analysis for Ba/F3-del19/T790M/C797S/L718M mutant cells. (a) Ba/F3-del19/T790M/C797S/L718M cells were treated with a single BI-4020 treatment and in combination with panitumumab for 6 h. (b) Ba/F3-del19/T790M/C797S/L718M cells were treated with a single BI-4020 treatment for 6 h or 24 hr.

Supplementary Figure 4

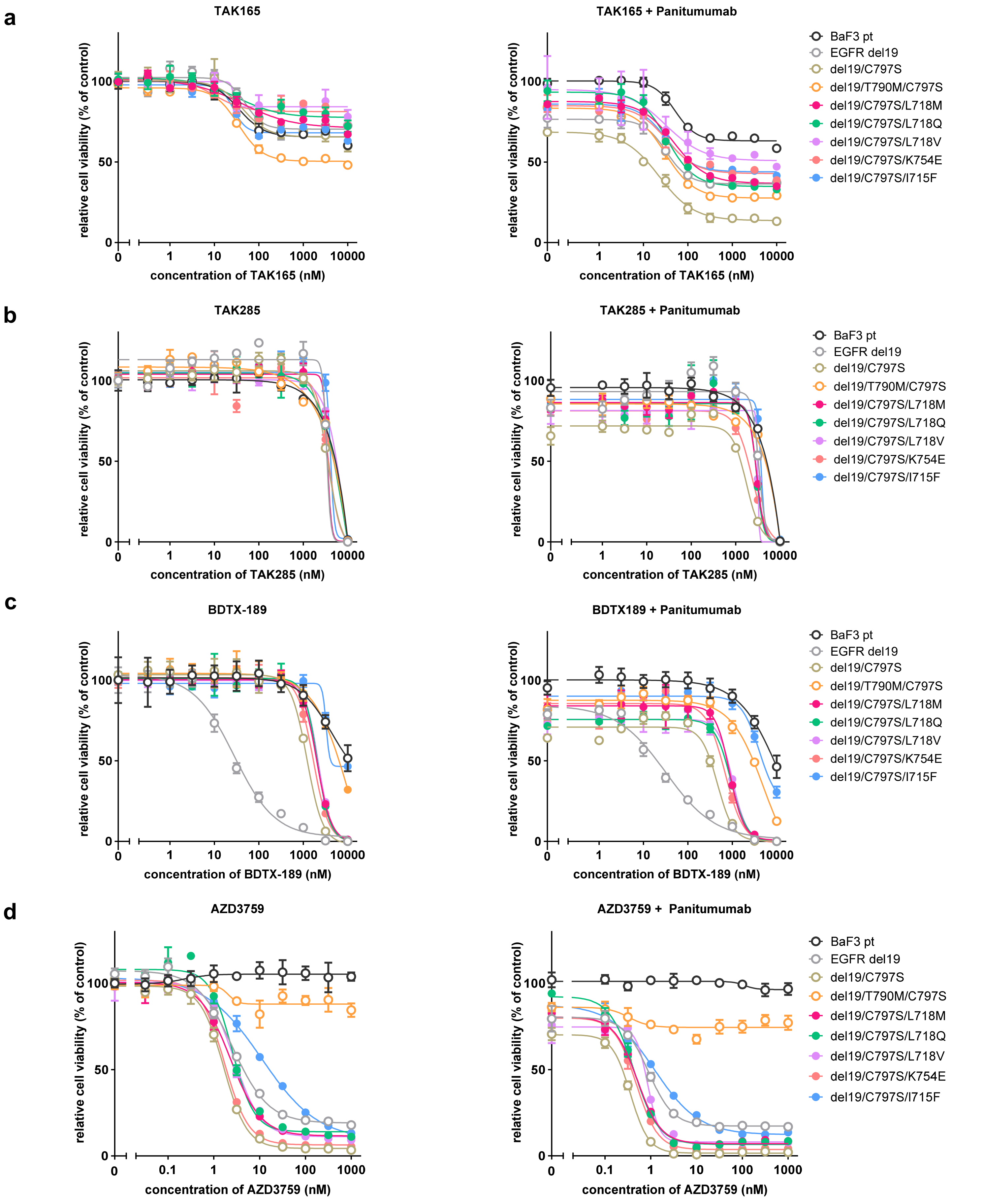

Supplementary Figure 4. CellTiter-Glo assay in the Ba/F3 ENU clone cells.
Each mutant cell was treated with TAK165 (a), TAK285 (b), BDTX-189 (c), AZD3759 (d), or in combination with panitumumab for 72 h. The viability was assessed by using a CellTiter-Glo assay. Experiments were repeated three times independently.

Supplementary Figure 5

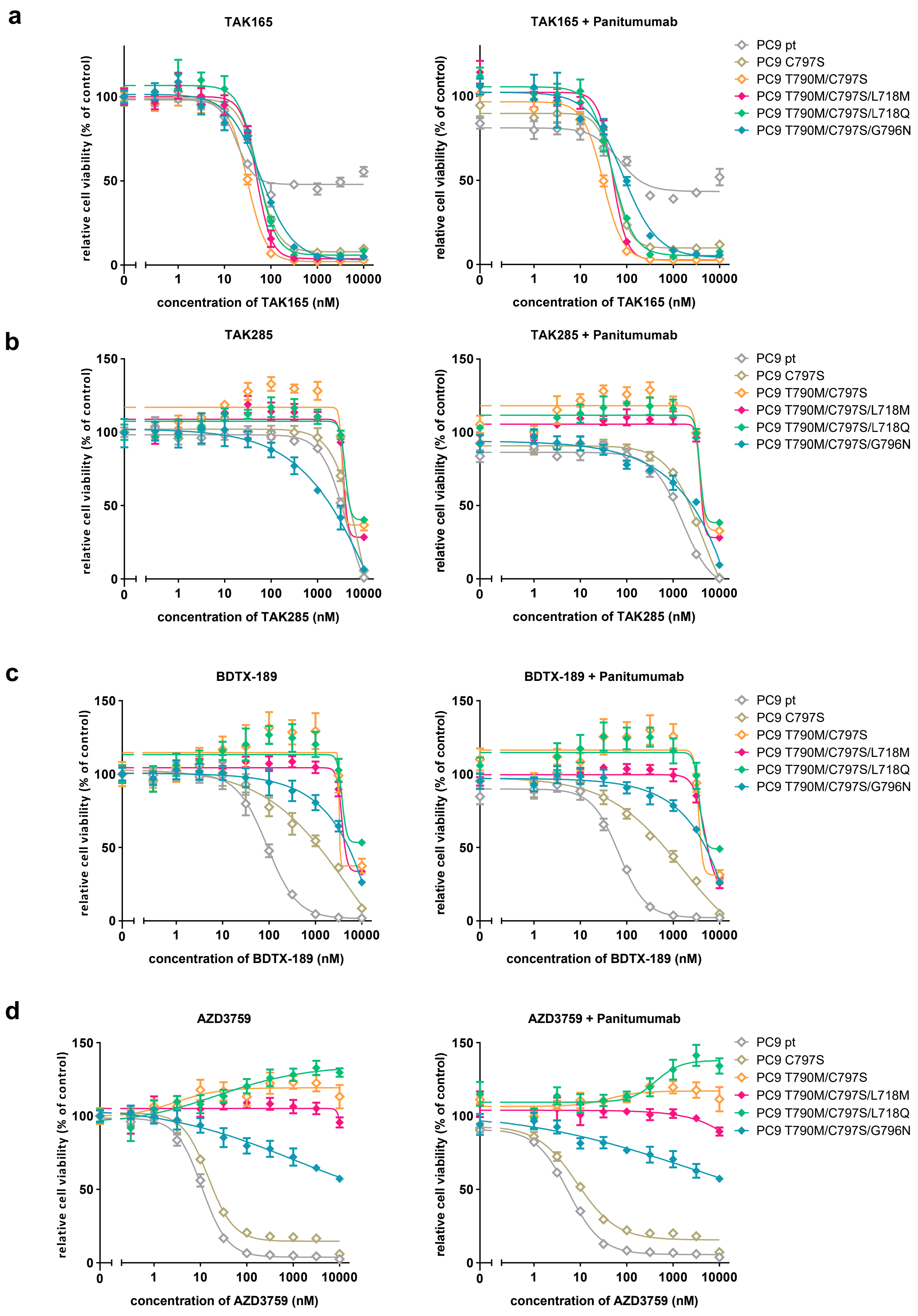

Supplementary Figure 5. CellTiter-Glo assay in EGFR mutant-expressing PC9 cells.
Each mutant cell was treated with TAK165 (a), TAK285 (b), BDTX-189 (c), AZD3759 (d), or in combination with panitumumab for 72 h. The viability was assessed by using a CellTiter-Glo assay. Experiments were repeated three times independently.

Supplementary Figure 6

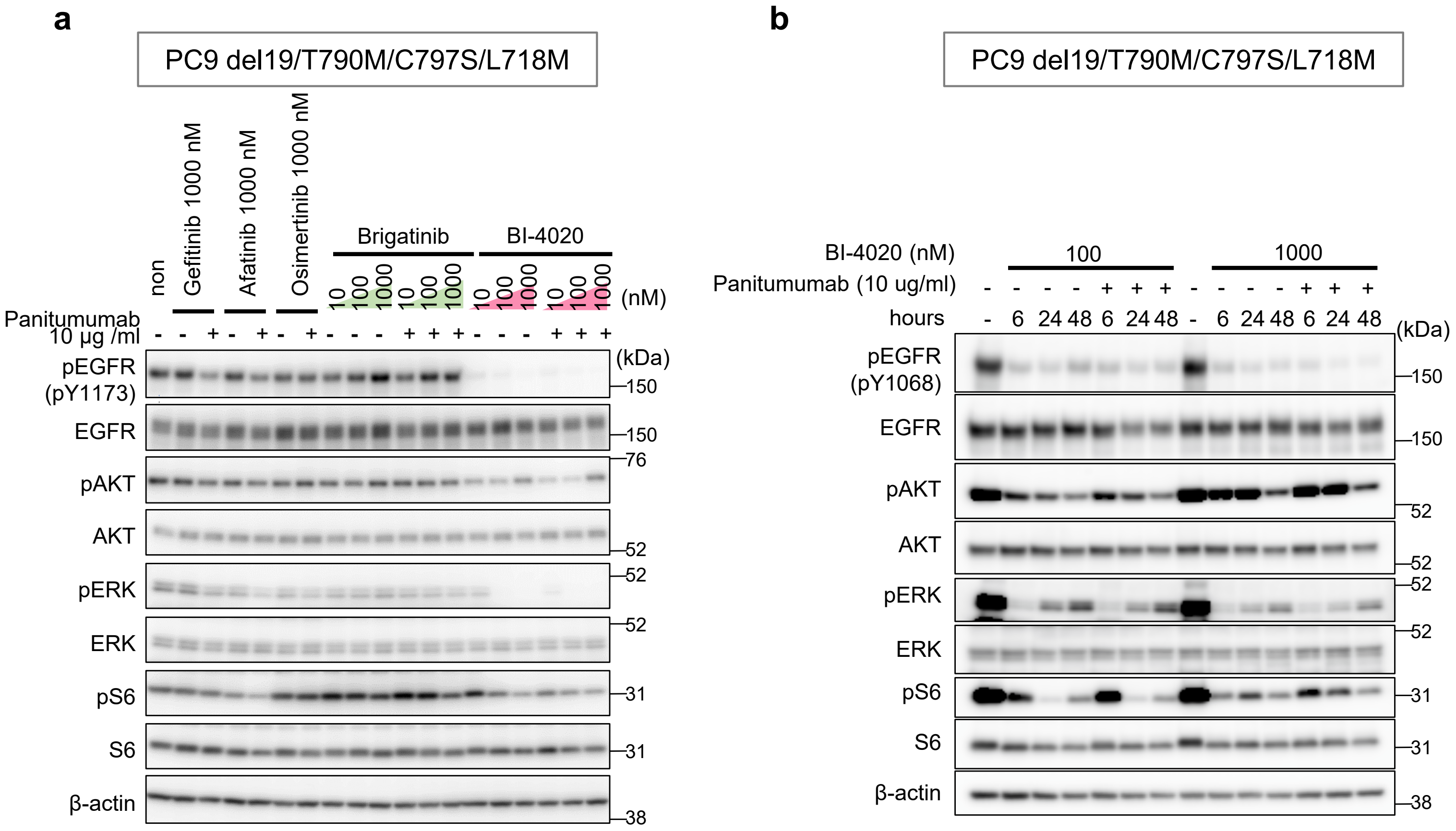

Supplementary Figure 6. Western blotting analysis for PC9-del19/T790M/C797S/L718M mutant cells.

(a) PC9-del19/T790M/C797S/L718M cells were treated with a single indicated EGFR-TKI treatment and in combination with panitumumab for 6h. This data is basically same experiments as Fig 4F but using anti phosoho-EGFR (pY1173) antibody. (b) PC9-del19/T790M/C797S/L718M cells were treated with a single BI-4020 treatment and in combination with panitumumab for 6, 24 or 48 hr. Cell lysates were analyzed by immunoblotting with the indicated antibodies.

Supplementary Figure 7

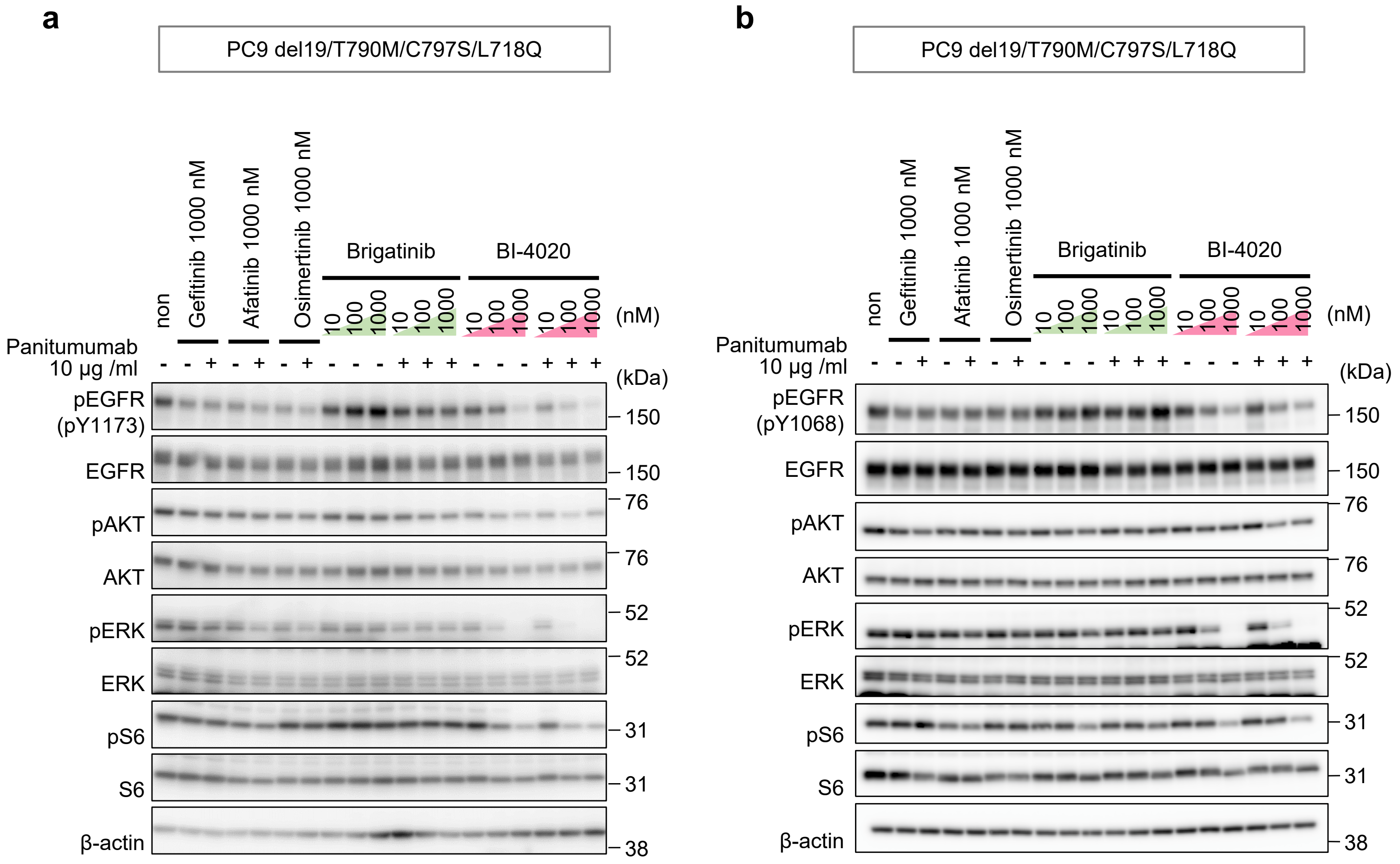

Supplementary Figure 7. Western blotting analysis for PC9-del19/T790M/C797S/L718Q mutant cells.
(a) PC9-del19/T790M/C797S/L718Q cells were treated with each concentration of drugs for 6 h. Collected cell lysates were analyzed by immunoblot with the indicated antibodies. (b) The same experiment was repeated and pEGFR (Tyr1068) was detected in B

Supplementary Figure 8

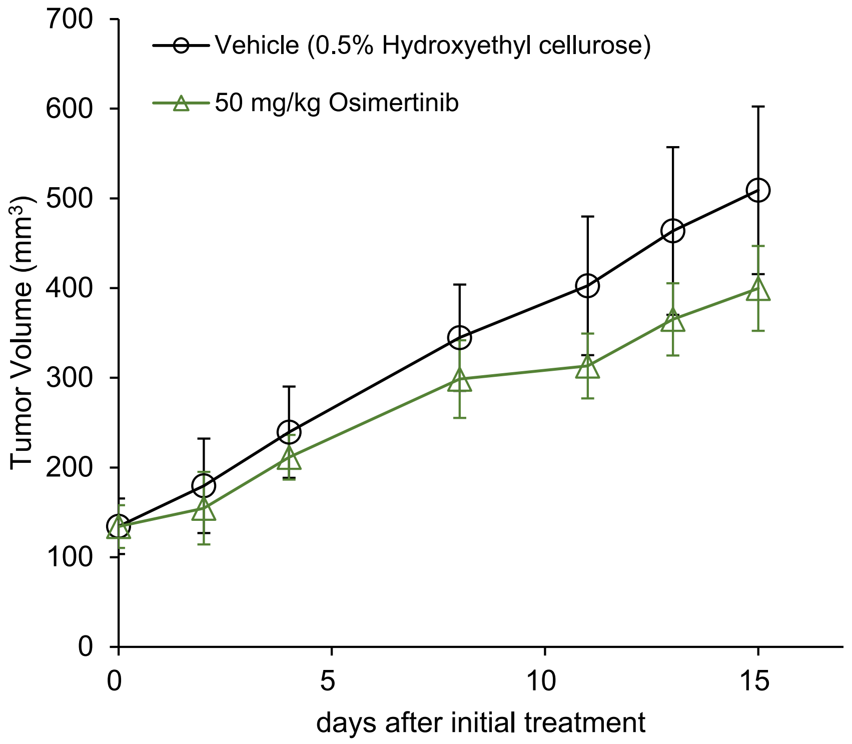

Supplementary Figure 8. PC9-del19/T790M/C797S/L718M were highly resistance to osimertinib in vivo. PC9-del19/T790M/C797S/L718M were subcutaneously injected into nude mice, and treated with the indicated doses of osimertinib.

Supplementary Figure 9

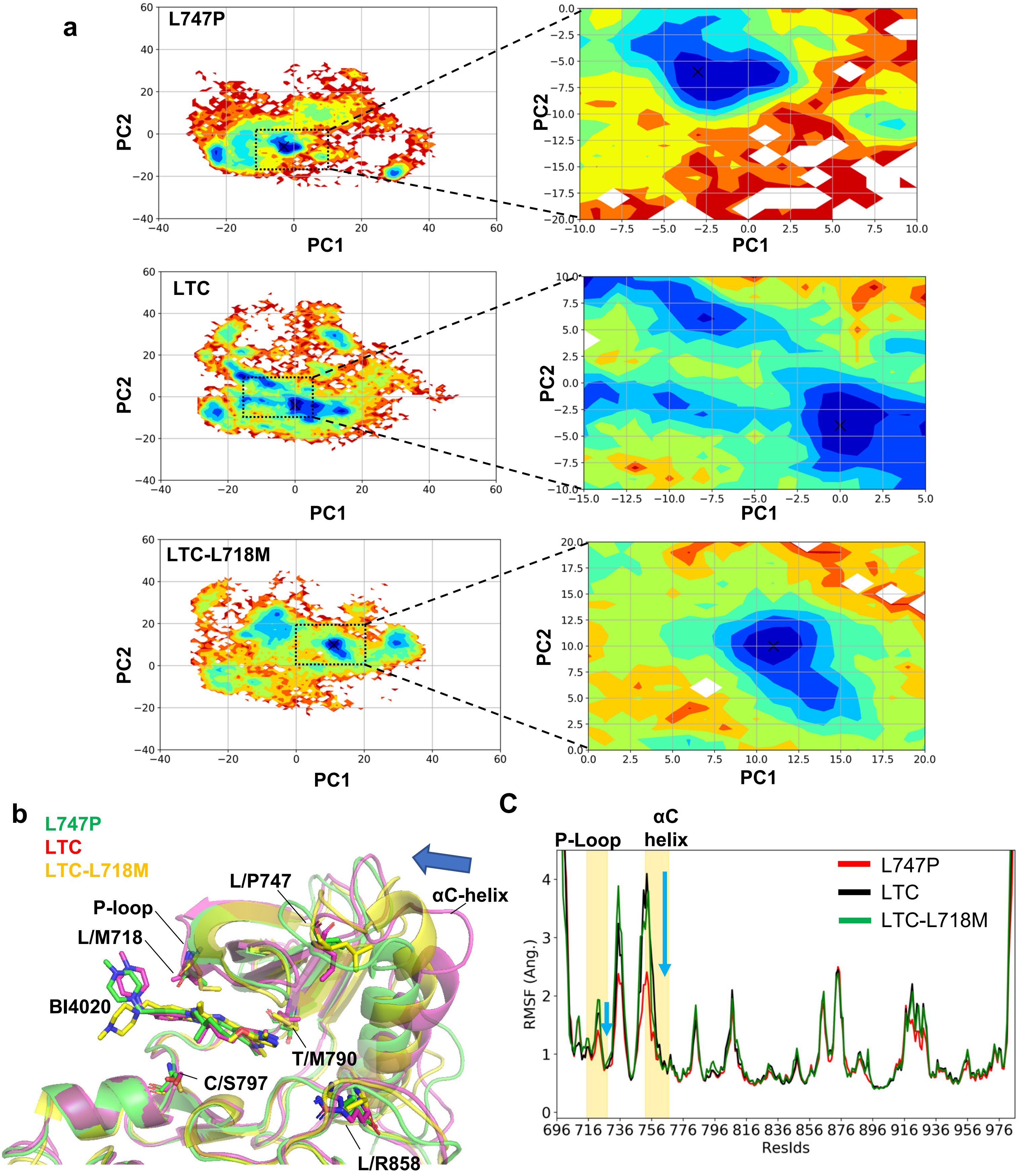

Supplementary Figure 9. Conformational dynamics of EGFR mutants complexed with BI4020 obtained from of microsecond-timescale MD simulation. (a) The free energy landscapes of the L747P, L858R/T790M/C797S, and L858R/T790M/C797S/L718M mutants as a function of the first two principal component (PC1 and PC2) calculated from Principal component analysis (PCA) of MD trajectories. Three independent 1 μs MD trajectories were acquired for each mutant, and after removing the overall translation and rotation of the protein, the covariance matrix was calculated using the Cartesian coordinates of the backbone Cα atoms to obtain the PC eigenvectors. Each landscape was calculated from the normalized probability distribution, according to $G(\text{PC1, PC2}) = -RT \ln (P(\text{PC1, PC2}) / \text{Pmax})$, where R is the gas constant, T is the absolute temperature, P is the probability density, and Pmax is the maximum probability density. The unit of the free energy is kcal/mol. An enlarged view around the most stable minimum (marked by X), which corresponds to Pmax, is shown in the right panel. (b) Structural comparison of the L747P, L7858R/T790M/C797S, and L858R/T790M/C797S/L718M mutants. Structures corresponding to the most stable minima in the free energy landscapes are shown. The protein backbone is represented by a ribbon diagram, and the side chains (L/M718, L/P747, T/M790, C/S797, and L/R858) and BI4020 are depicted as sticks. An orientational change in the αC-helix upon the L747P mutation is indicated by blue arrows. (c) Root-mean-square fluctuation (RMSF) of backbone Cα atoms. RMSF values were calculated using the MD trajectories of 1 μs × 3. P-loop and αC-helix regions are highlighted in yellow. Conformational flexibility of these regions in the L747P mutant is lower than that in the L858R/T790M/C797S and L858R/T790M/C797S/L718M mutants, as indicated by blue arrows.

Supplementary Figure 10

Original uncropped immunoblot images for Figure 3f

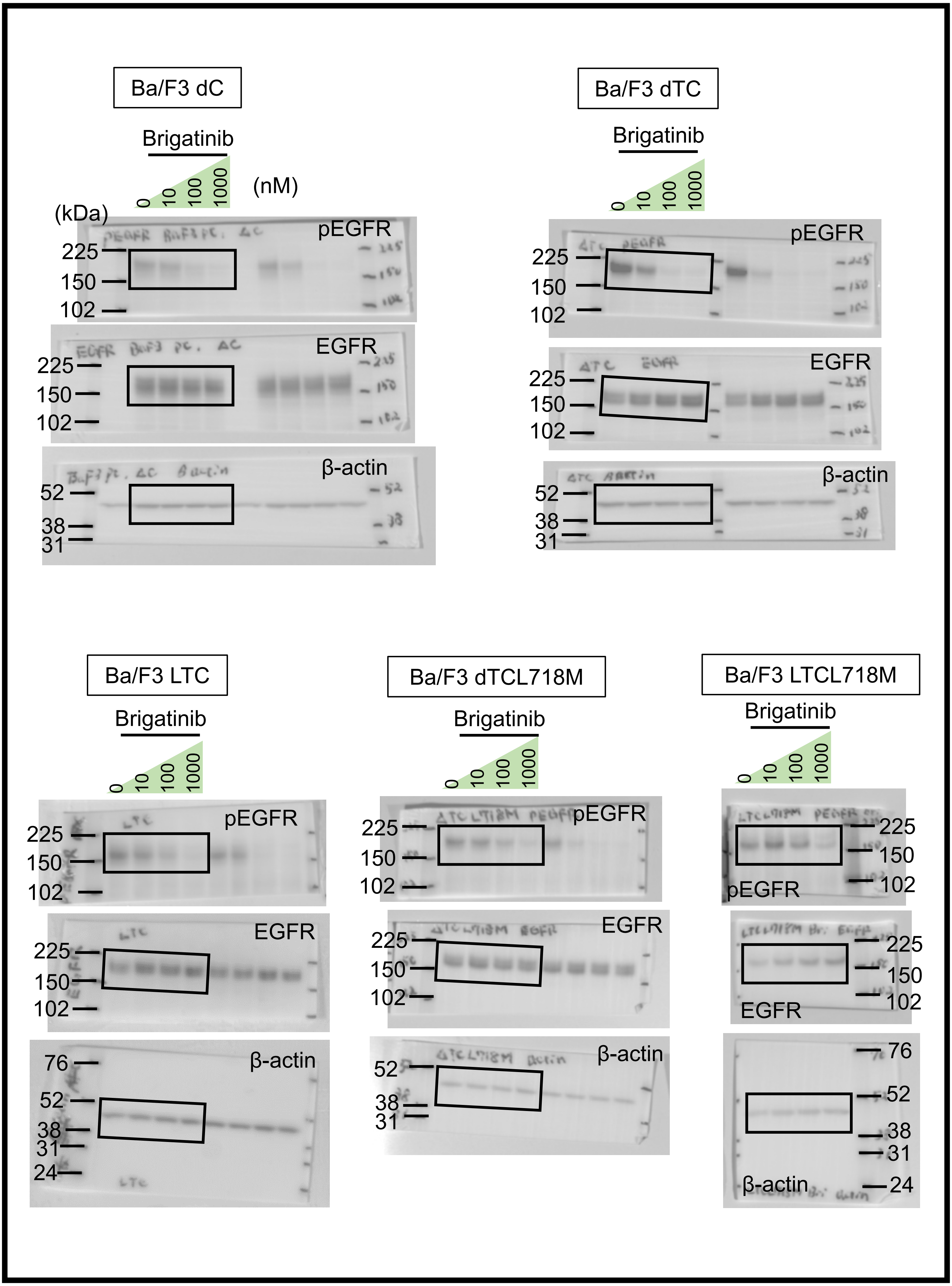

Supplementary Figure 10

Original uncropped immunoblot images for Figure 3f

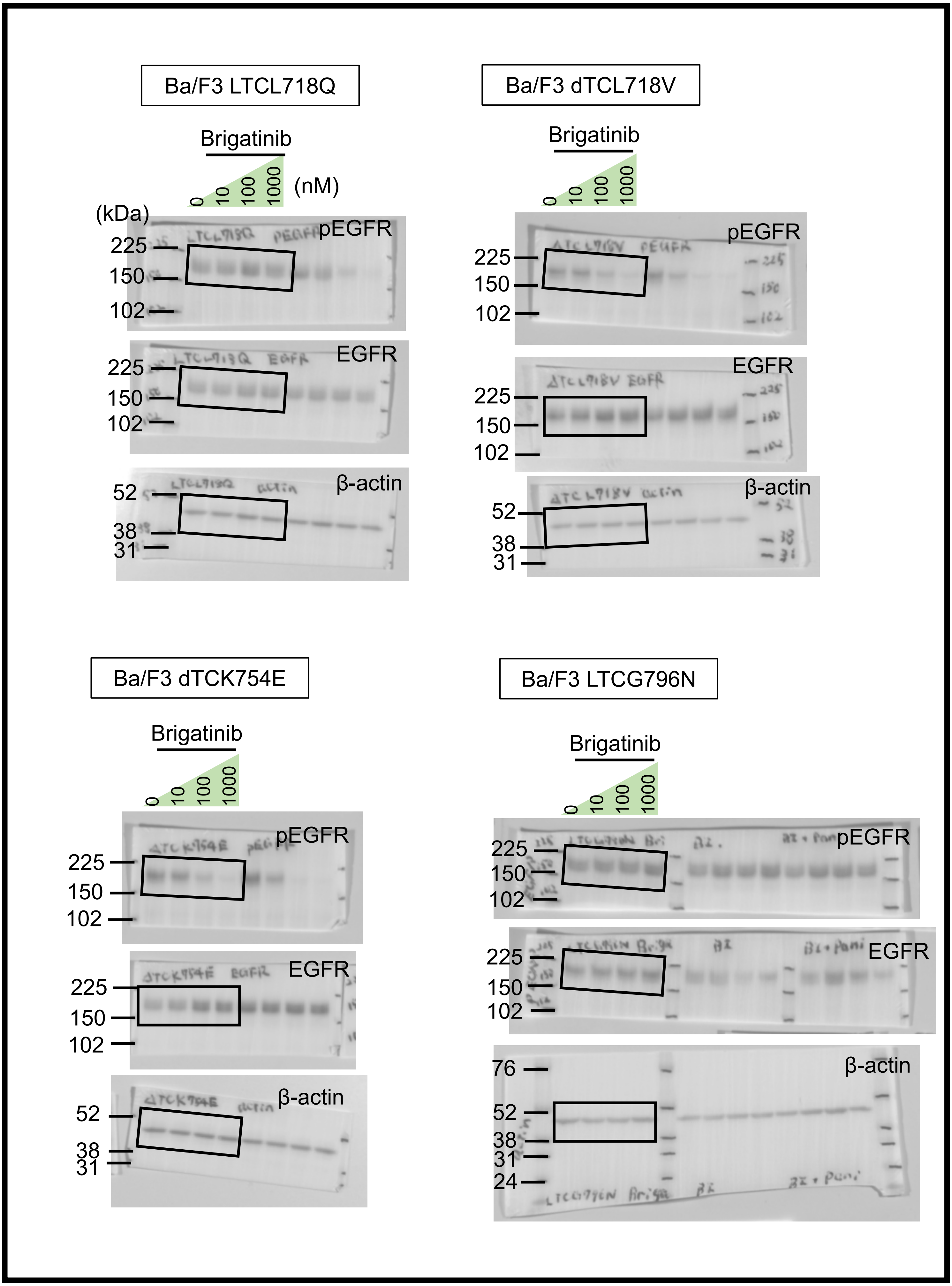

Supplementary Figure 10

Original uncropped immunoblot images for Figure 4c

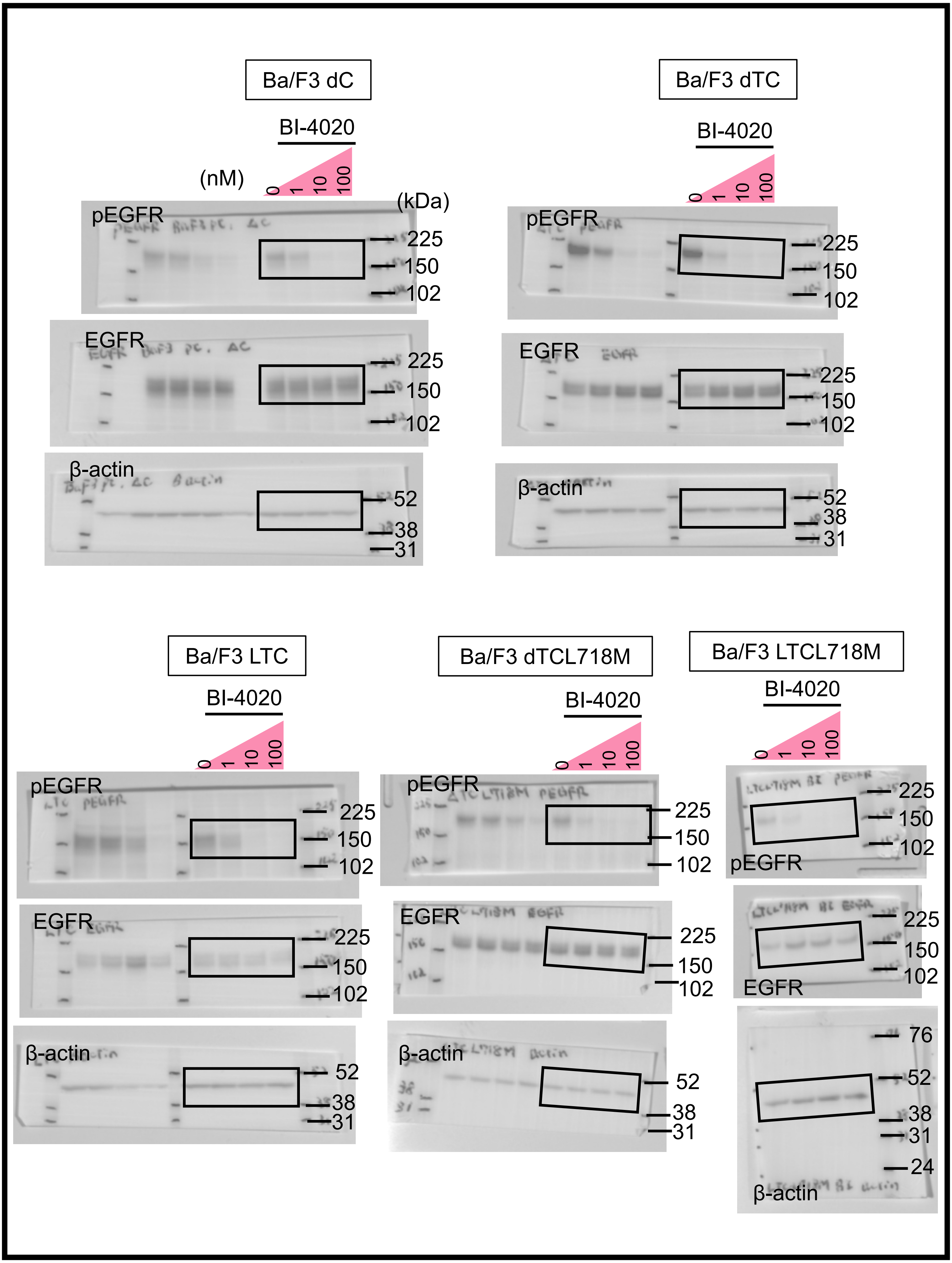

Supplementary Figure 10

Original uncropped immunoblot images for Figure 4c

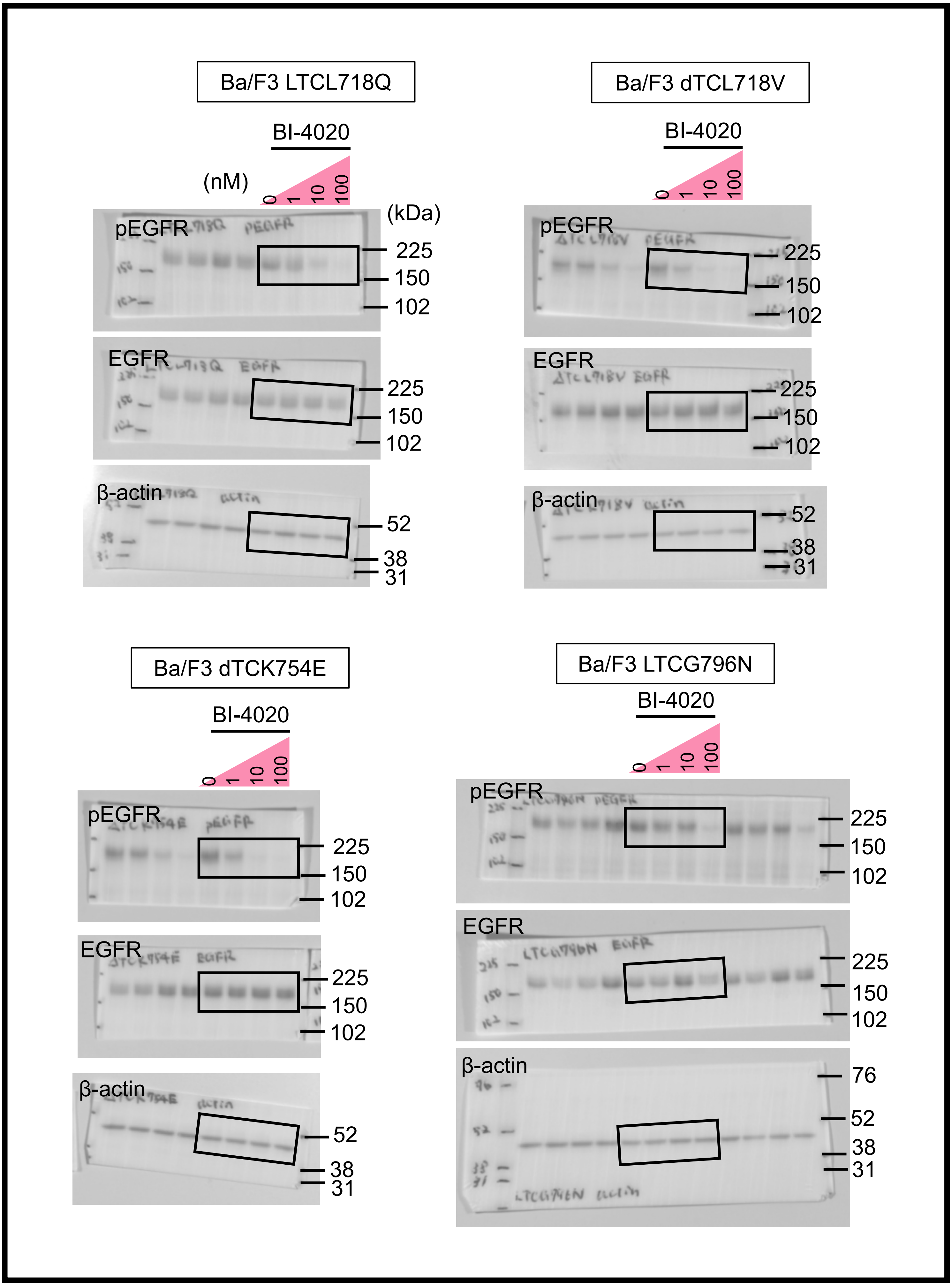

Supplementary Figure 10

Original uncropped immunoblot images for Figure 4f

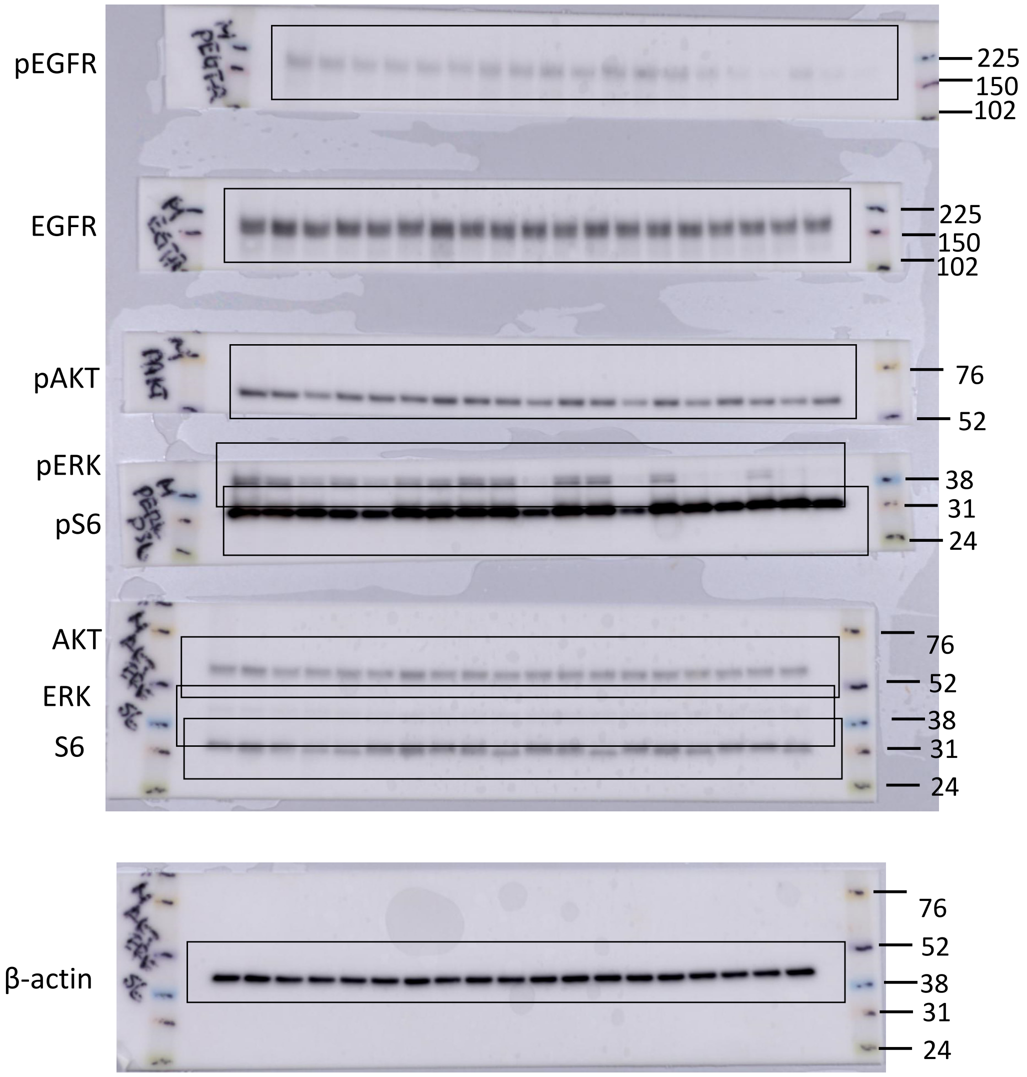

In the next page, uncropped images of different exposure time are indicated.

Supplementary Figure 10

Original uncropped immunoblot images for Figure 4f

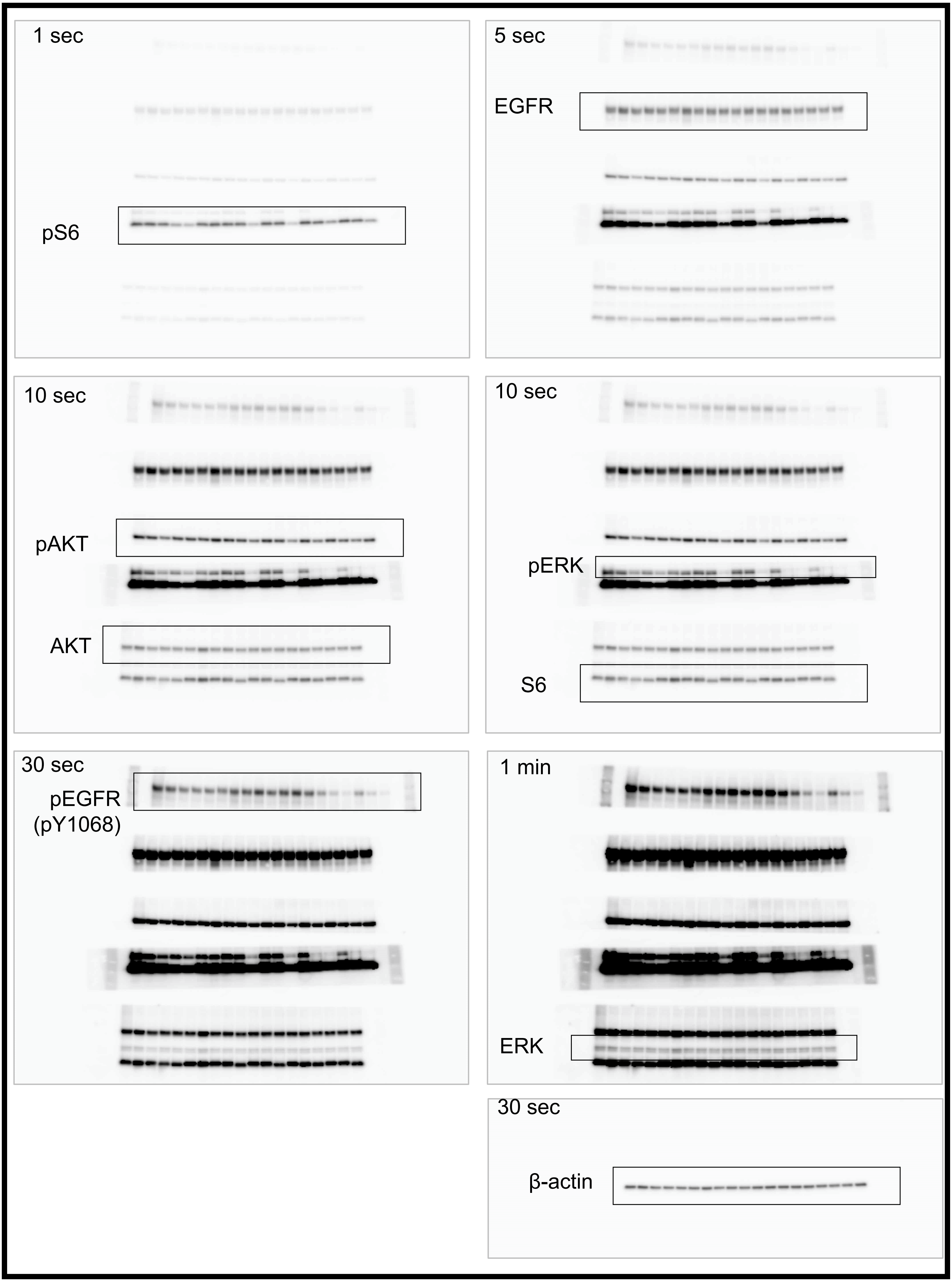

Supplementary Figure 10

Original uncropped immunoblot images for Figure 6c

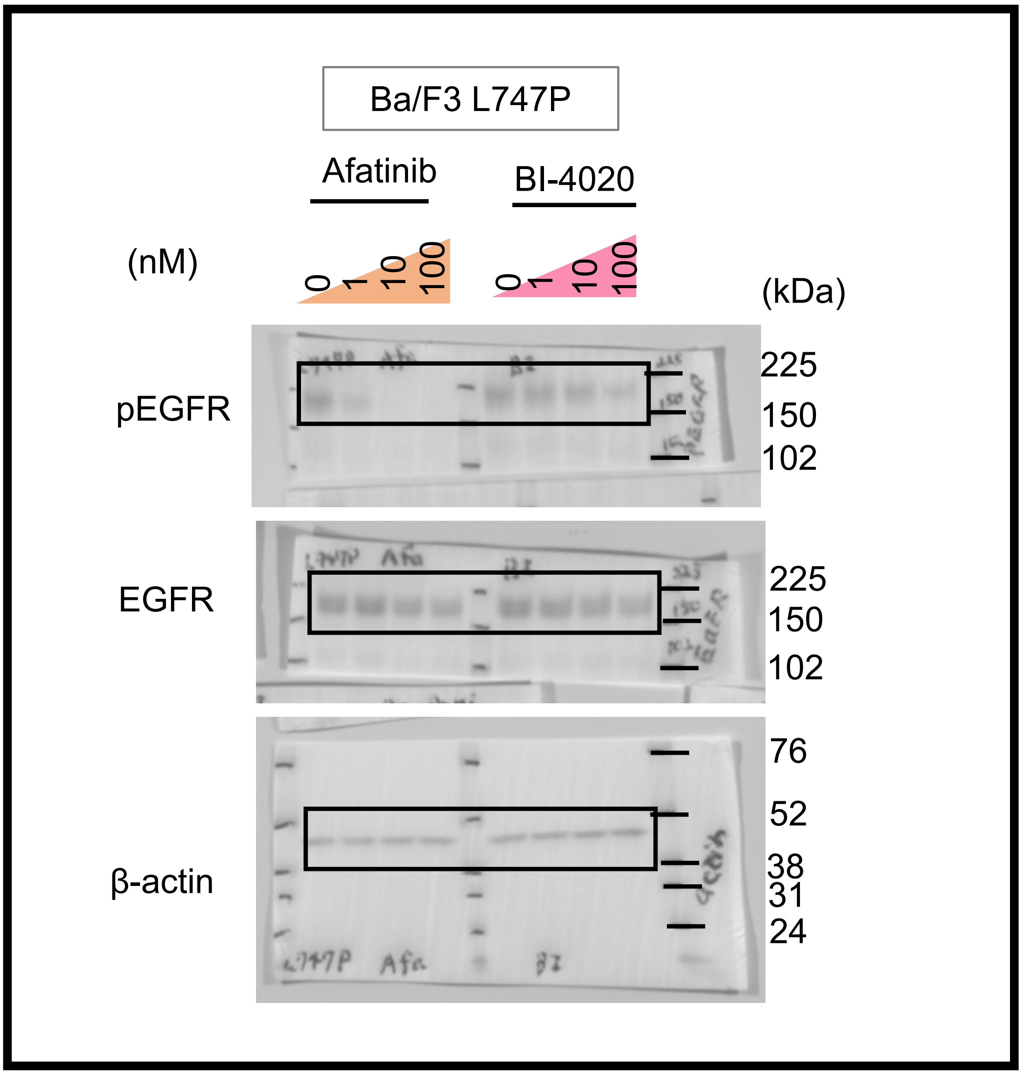

Supplementary Table 1. Crystallographic statistics of the EGFR-T790M/C797S and Brigatinib complex

**Data collection**	
Space group	$P2_12_12_1$
Cell dimensions	
a, b, c (Å)	50.1, 90.1, 165.3
α, β, γ (°)	90, 90, 90
Resolution range (Å)	47.95–3.40 (3.61–3.40)
Redundancy	7.2 (7.3)
Completeness (%)	99.9 (100)
$I/\sigma(I)$	11.2 (1.8)
R_{meas}	0.209 (1.436)
$CC_{1/2}$	0.996 (0.764)
**Refinement**	
Resolution range (Å)	47.95–3.40 (3.56–3.40)
No. of reflections	10,814
R-factor/free *R*-factor*	0.181 / 0.256
No. of atoms	
Protein	4,768
Ligand	40
Water	0
Average B values ($Å^2$)	
Protein	103.6
Ligand	95.5
Water	-
Root-mean-square deviations	
Bond lengths (Å)	0.010
Bond angles (°)	1.246
Ramachandran plot (%)	87.8, 12.0, 0.2, 0.0

All numbers in parentheses refer to the highest resolution shell statistics.

*Free *R* factor was calculated for 10% of randomly selected reflections excluded from refinement.

Supplementary Table2: Detailed materials information

REAGENT or RESOURCE	SOURCE	IDENTIFIER
Antibodies		
Phospho-EGF Receptor (Tyr1173) (53A5) Rabbit mAb	Cell Signaling Technology	Cat# 4407, RRID: AB_331795
EGFR (phospho Tyr1068) antibody	GeneTex	Cat# GTX132810, RRID: AB_2886752
EGF Receptor (D38B1) XP® Rabbit mAb	Cell Signaling Technology	Cat# 4267, RRID: AB_2864406
Phospho-Akt (Ser473) (D9E) XP® Rabbit mAb	Cell Signaling Technology	Cat# 4060, RRID: AB_2315049
Akt (pan) (C67E7) Rabbit mAb	Cell Signaling Technology	Cat# 4691, RRID: AB_915783
p44/42 MAP kinase (phosphorylated Erk1/2)	Cell Signaling Technology	Cat# 9101, RRID: AB_331646
p44/42 MAPK (Erk1/2) Antibody	Cell Signaling Technology	Cat# 9102, RRID: AB_330744
Phospho-S6 Ribosomal Protein (Ser240/244) (D68F8) XP Rabbit mAb	Cell Signaling Technology	Cat# 5364, RRID: AB_10694233
Rabbit Anti-S6 Ribosomal Protein Monoclonal Antibody, Unconjugated, Clone 5G10	Cell Signaling Technology	Cat# 2217, RRID: AB_331355
Monoclonal Anti-Actin, alpha-Smooth Muscle antibody produced in mouse	Sigma-Aldrich	Cat# A5228, RRID: AB_262054
Donkey Anti-Rabbit IgG, Whole Ab ECL Antibody, HRP Conjugated	Cytiva	Cat# NA934, RRID: AB_772206
Sheep Anti-Mouse IgG - Horseradish Peroxidase	Cytiva	Cat# NA931, RRID: AB_772210
Chemicals, peptides, and recombinant proteins		
Brigatinib	Biochempartner	BCP06648
Cetuximab	Merck	205923-56-4
Panitumumab	Takeda Pharma.	339177-26-3

Necitumumab	Eli Lilly	N/A
Osimertinib	Selleck	S7297
Gefitinib	LC laboratories	G-4408
Afatinib	Selleck	S1011
BI-4020	Med Chem Express	HY-129550
BI-4020 for in vivo study (Figure 5)	Boehringer Ingelheim	N/A
TAK165	Med Chem Express	HY-13501
TAK285	Med Chem Express	HY-15196
BDTX-189	Med Chem Express	HY-136789
AZD3759	Med Chem Express	HY-18750
N-ethyl-N-nitrosourea	Sigma Aldrich	N3385
Critical commercial assays		
CellTiter-Glo assay	Promega	G7573
ADP-Glo assay	Promega	V6930
BCA Protein Assay Reagent	Thermo Fisher Scientific	A53225
Deposited data		
EGFR-T790M/C797S structure with Brigatinib	This paper	PDB: 8H7X
Experimental models: Cell lines		
Mouse: Ba/F3	RIKEN BioResource Center	RCB4476
Human: PC9	Dr. Kazuto Nishio	N/A
Human: A431	ATCC	CRL-1555
Human: A549	NCI-60	N/A
Human: 293FT	Invitrogen, Carlsbad, CA, USA	R70007
Human: MGH121	Dr. Jeffrey.A. Engelman	N/A
Experimental models: Organisms/strains		
Mouse: Balb-c nu/nu	Charles River Laboratories	N/A
Oligonucleotides		

EGFR L718M F:5’-AATTCAAAAAGATCAAAGTGATGGGCTCCGGTGCG-3’	Hokkaido System Science	N/A
EGFR L718M R: 5’-CGCACCGGAGCCCATCACTTTGATCTTTTTGAATT-3’	Hokkaido System Science	N/A
EGFR L718Q F: 5’-AAAAAGATCAAAGTGCAGGGCTCCGGTGCGTTC-3’	Hokkaido System Science	N/A
EGFR L718Q R: 5’-GAACGCACCGGAGCCCTGCACTTTGATCTTTTT-3’	Hokkaido System Science	N/A
EGFR L718V F: 5’-AATTCAAAAAGATCAAAGTGGTGGGCTCCGGTGCG-3’	Hokkaido System Science	N/A
EGFR L718V R: 5’-CGCACCGGAGCCCACCACTTTGATCTTTTTGAATT-3’	Hokkaido System Science	N/A
EGFR K754E F: 5’-GCTATCAAGACATCTCCGGAAGCCAACAAGGAAATCC-3’	Hokkaido System Science	N/A
EGFR K754E R: 5’-GGATTTCCTTGTTGGCTTCCGGAGATGTCTTGATAGC-3’	Hokkaido System Science	N/A
Recombinant DNA		
pLenti6.3/V5-DEST™ Gateway™ Vector Kit	Thermo Fisher Scientific	V53306
Software and algorithms		
GraphPad Prism version 7.0.4	GraphPad software	https://www.graphpad.com:443/
